# Pan-cancer chromatin analysis of the human vtRNA genes uncovers their association with cancer biology

**DOI:** 10.12688/f1000research.28510.2

**Published:** 2021-06-09

**Authors:** Rafael Sebastián Fort, María Ana Duhagon

**Affiliations:** 1 Laboratorio de Interacciones Moleculares, Facultad de Ciencias, Universidad de la República, Montevideo, Montevideo, 11400, Uruguay; 2 Depto. de Genómica, Instituto de Investigaciones Biológicas Clemente Estable, Montevideo, Montevideo, 11600, Uruguay; 3 Depto. de Genética, Facultad de Medicina, Universidad de la República, Montevideo, Montevideo, 11400, Uruguay

**Keywords:** vault RNA, vtRNA1-1, vtRNA1-2, vtRNA1-3, vtRNA2-1, nc886, mir-886, cancer, TCGA, DNA methylation, chromatin accessibility

## Abstract

**Background:** The vault RNAs (vtRNAs) are a class of 84-141-nt eukaryotic non-coding RNAs transcribed by RNA polymerase III, associated to the ribonucleoprotein complex known as vault particle. Of the four human vtRNA genes, vtRNA1-1, vtRNA1-2 and vtRNA1-3, clustered at locus 1, are integral components of the vault particle, while vtRNA2-1 is a more divergent homologue located in a second locus. Gene expression studies of vtRNAs in large cohorts have been hindered by their unsuccessful sequencing using conventional transcriptomic approaches.

**Methods:** VtRNA expression in The Cancer Genome Atlas (TCGA) Pan-Cancer cohort was estimated using the genome-wide DNA methylation and chromatin accessibility data (ATAC-seq) of their genes as surrogate variables. The association between vtRNA expression and patient clinical outcome, immune subtypes and transcriptionally co-regulated gene programs was analyzed in the dataset.

**Results:** VtRNAs promoters are enriched in transcription factors related to viral infection. VtRNA2-1 is likely the most independently regulated homologue. VtRNA1-1 has the most accessible chromatin, followed by vtRNA1-2, vtRNA2-1 and vtRNA1-3. VtRNA1-1 and vtRNA1-3 chromatin status does not significantly change in cancer tissues. Meanwhile, vtRNA2-1 and vtRNA1-2 expression is widely deregulated in neoplastic tissues and its alteration is compatible with a broad oncogenic role for vtRNA1-2, and both tumor suppressor and oncogenic functions for vtRNA2-1. Yet, vtRNA1-1, vtRNA1-2 and vtRNA2-1 promoter DNA methylation predicts a shorter patient overall survival cancer-wide. In addition, gene ontology analyses of vtRNAs co-regulated genes identify a chromosome regulatory domain, epithelial differentiation, immune and thyroid cancer gene sets for specific vtRNAs. Furthermore, vtRNA expression patterns are associated with cancer immune subtypes and vtRNA1-2 expression is positively associated with cell proliferation and wound healing.

**Conclusions:** Our study presents the landscape of vtRNA chromatin status cancer-wide, identifying co-regulated gene networks and ontological pathways associated with the different vtRNA genes that may account for their diverse roles in cancer.

## Abbreviations

vtRNA            vault RNA

TCGA             The Cancer Genome Atlas

ATAC-seq        Assay for Transposase Accessible Chromatin with high-throughput sequencing

NoMe-seq        Nucleosome Occupancy and Methylome sequencing

OG                  Oncogene

TSG                Tumor Suppressor Gene

TCGA             The Cancer Genome Atlas

TSS                 Transcription Start Site

Ave                  Average

SD                   Standard Deviation 

ACC                Adrenocortical carcinoma

BLCA              Bladder Urothelial Carcinoma

BRCA              Breast invasive carcinoma

CESC              Cervical squamous cell carcinoma and endocervical adenocarcinoma

CHOL             Cholangiocarcinoma

COAD            Colon adenocarcinoma

DLBC             Lymphoid Neoplasm Diffuse Large B-cell Lymphoma

ESCA             Esophageal carcinoma

GBM              Glioblastoma multiforme

HNSC             Head and Neck squamous cell carcinoma

KICH              Kidney Chromophobe

KIRC              Kidney renal clear cell carcinoma

KIRP              Kidney renal papillary cell carcinoma

LGG               Brain Lower Grade Glioma

LIHC              Liver hepatocellular carcinoma

LUAD             Lung adenocarcinoma

LUSC             Lung squamous cell carcinoma

MESO            Mesothelioma

OV                 Ovarian serous cystadenocarcinoma

PAAD             Pancreatic adenocarcinoma

PCPG             Pheochromocytoma and Paraganglioma

PRAD             Prostate adenocarcinoma

READ             Rectum adenocarcinoma

SARC             Sarcoma

SKCM            Skin Cutaneous Melanoma

STAD             Stomach adenocarcinoma

TGCT            Testicular Germ Cell Tumors

TGCT            Testicular Germ Cell Tumors

THCA            Thyroid carcinoma

THYM            Thymoma

UCEC             Uterine Corpus Endometrial Carcinoma

UCS               Uterine Carcinosarcoma

UVM              Uveal Melanoma

## Introduction

The vault RNAs (vtRNAs) are a class of eukaryotic middle size non-coding RNAs (84-141 nt) transcribed by the RNA polymerase III, which associates to the vault particle (<5 % of the total mass of the vault particle) ( Boivin *et al.*, 2019; Kedersha & Rome, 1986; Kedersha *et al.*, 1991). Although this particle is the largest cellular ribonucleoprotein complex, its function remains scarcely understood. Most mammals have a single vtRNA gene (i.e., mouse and rat), but the human genome has three copies, annotated as vtRNA1-1 (98bp), vtRNA1-2 and vtRNA1-3 (88bp) genes, which are located in the chromosome region 5q31.3 (locus vtRNA1) and code for the RNAs that are integral components of the vault particle. Lately, a transcript initially annotated as the human microRNA precursor hsa-mir-886 ( Landgraf *et al.*, 2007), was re-classified as another human vtRNA homologue and consequently renamed as vtRNA2-1 ( Stadler *et al.*, 2009). VtRNA2-1 is also located in chromosome 5 (5q31.1, locus vtRNA2) at 0.5Mb distance from the vtRNA1 cluster, where is placed between SMAD5 and TGFBI genes in an antisense direction. Existing evidence indicates that despite being a duplication of the vtRNAs of locus 1, the vtRNA2-1 is neither associated with the vault particle nor co-regulated with the vtRNA1 locus ( Lee *et al.*, 2011; Stadler *et al.*, 2009). Nevertheless, the realization that only 5–20% of all the cellular vtRNA transcripts are associated to the vault particle suggests additional roles for the majority of vtRNA transcripts, independent of the vault particle ( Kickhoefer *et al.*, 1998; Nandy *et al.*, 2009; van Zon *et al.*, 2001). The vtRNA3-1P and vtRNA2-2P are two additional vault RNA genes situated in chromosome X and 2 respectively, but were classified as a pseudogenes due to the absence of expression in cell lines and the presence of silencing mutations at the promoter ( Büscher *et al.*, 2020; van Zon *et al.*, 2001).

Most of the current knowledge of vtRNA function focuses on vtRNA1-1 and vtRNA2-1 roles in viral infection and cancer. Functional studies found that vtRNA1-1 and vtRNA1-2 (but not vtRNA1-3) can interact directly with drugs as mitoxantrone, doxorubicin and etoposide ( Gopinath *et al.*, 2005; Gopinath *et al.*, 2010; Mashima *et al.*, 2008). In addition, p62 protein interacts with vtRNA1-1 and inhibits the p62 dependent autophagy *in vitro* and *in vivo* ( Horos *et al.*, 2019). The vault RNAs have also been linked to native immune response, because of their strong upregulation during the infection of Influenza A virus (IAV) and Epstein-Barr virus (EBV) ( Amort *et al.*, 2015; Li *et al.*, 2015; Nandy *et al.*, 2009). These studies proposed a viral activation of host vtRNAs as a mechanism to maintain the inhibition of PKR signaling pathway, representing a viral strategy to circumvent host innate immunity.

Initial reports about vtRNA2-1 (locus vtRNA2) revealed that it is abundant in normal tissues while lowly expressed in cancer cell lines from different tissue origin (breast, melanoma, cervix, lung, oral and prostate), which is consistent with a tumor suppressive role in cancer ( Lee *et al.*, 2011; Treppendahl *et al.*, 2012). Moreover, vtRNA2-1 was proposed as a new type of ncRNA functioning as a tumor suppressor gene (TSG) that inhibits PKR, and was consequently renamed as “nc886” ( Golec *et al.*, 2019; Jeon *et al.*, 2012a; Jeon *et al.*, 2012b; Lee *et al.*, 2011). Then, its anti-proliferative and TSG function was described in prostate ( Aakula *et al.*, 2016; Fort *et al.*, 2018; Ma *et al.*, 2020), skin ( Lee *et al.*, 2019a), gastric ( Lee *et al.*, 2014b) esophageal ( Im *et al.*, 2020; Lee *et al.*, 2014a) and cholangiocarcinoma cells ( Kunkeaw *et al.*, 2012). Conversely, a pro-proliferative and anti-apoptotic oncogenic role was proposed for vtRNA2-1 in renal ( Lei *et al.*, 2017), ovarian ( Ahn *et al.*, 2018), thyroid ( Lee *et al.*, 2016), cervical ( Li *et al.*, 2017) and endometrial ( Hu *et al.*, 2017) tissues. A recent finding proposing a vtRNA2-1/PKR loss mediated doxorubicin cytotoxicity introduced a novel view about its contribution to chemotherapy response ( Kunkeaw *et al.*, 2019). In addition, a potential TSG role has been put forward for vtRNA1-3 in Myelodysplastic syndrome (MDS) ( Helbo *et al.*, 2015).

Apart from their role as full length RNAs, vtRNAs are precursors of small RNAs. Indeed, small RNAs with microRNA like function were demonstrated to derive from vtRNA1-1 (svRNAs) and to repress the expression CYP3A4, a key enzyme of drug metabolism ( Meng *et al.*, 2016; Persson *et al.*, 2009). VtRNA2-1 has also been shown to generate small RNAs that could act like microRNAs in different tissues, serving both as TSG in prostate ( Aakula *et al.*, 2016; Fendler *et al.*, 2011; Fort *et al.*, 2020), bladder ( Nordentoft *et al.*, 2012), breast ( Tahiri *et al.*, 2014), colon ( Yu *et al.*, 2011), lung ( Bi *et al.*, 2014; Cao *et al.*, 2013; Gao *et al.*, 2011; Shen *et al.*, 2018) and thyroid cancers ( Dettmer *et al.*, 2014; Xiong *et al.*, 2011) and as oncogene (OG) in renal ( Yu *et al.*, 2014), colorectal ( Schou *et al.*, 2014) and esophagus cancer ( Okumura *et al.*, 2016) through the repression of specific mRNA transcripts in human cancer.

Different lines of evidence revealed that the vtRNA transcription can be controlled by promoter DNA methylation. The epigenetic control of vtRNA2-1 is complex and owns clinical relevance in several solid tumors including breast, lung, colon, bladder, prostate, esophagus, hepatic and stomach cancer ( Cao *et al.*, 2013; Fort *et al.*, 2018; Lee *et al.*, 2014a; Lee *et al.*, 2014b; Romanelli *et al.*, 2014; Treppendahl *et al.*, 2012; Yu *et al.*, 2020). Intriguing aspects of the epigenetic regulation of vtRNA2-1 locus comprise its dependence on the parental origin of the allele ( Joo *et al.*, 2018; Paliwal *et al.*, 2013), and its sensitivity to the periconceptional environment (( Silver *et al.*, 2015) and subsequent independent studies ( Richmond *et al.*, 2018; van Dijk *et al.*, 2018)). In addition, vtRNA1-3 promoter methylation was associated with significantly poor outcome in lower risk myelodysplastic syndrome patients ( Helbo *et al.*, 2015).

Currently, transcriptomic sequencing is the benchmark technique to study global RNA expression ( Stark *et al.*, 2019). Nonetheless, some classes of RNAs are elusive to the standard transcriptomics due to their stable RNA structure and the presence of modified bases or ends, which impair the cDNA synthesis and/or adapter ligation during the sequencing library preparation ( Sendler *et al.*, 2011; Zheng *et al.*, 2015). The vtRNAs belong to this group due to their conserved stable stem/hairpin loop secondary structure. The latter, together with the lack of sequencing of 40–200bp-long RNAs in the conventional transcriptomic studies, which are mostly intended for small and long RNAs, probably delayed their study in comparison with other regulatory RNAs. In fact, the 50–300 nucleotides long RNAs are considered the black hole of RNA biology ( Boivin *et al.*, 2018; Steitz, 2015). Nonetheless, since chromatin accessibility plays a critical role in the regulation of gene expression, at some extent the transcription of an RNA can be inferred from the chromatin status of its promoter ( Corces *et al.*, 2018; Duren *et al.*, 2017; Liu *et al.*, 2018; Park *et al.*, 2017b). Since mounting evidence has shown that vtRNAs expression is tightly controlled by chromatin accessibility, dependent on nucleosome positioning and promoter DNA methylation ( Ahn *et al.*, 2018; Fort *et al.*, 2018; Helbo *et al.*, 2015; Helbo *et al.*, 2017; Lee *et al.*, 2014a; Lee *et al.*, 2014b; Park *et al.*, 2017a; Park *et al.*, 2017b; Sallustio *et al.*, 2016; Treppendahl *et al.*, 2012), the chromatin structure is a suitable surrogate marker of vtRNA transcription and could be used as a proxy of their expression.

The expression, regulation, and role of the four human vtRNAs in normal and disease conditions are still poorly understood, and available knowledge indicates that they hold diverse tissue specific activities. The availability of genomic data of large sets of human tissues provided by The Cancer Genome Atlas (TCGA), allows the study of the human vtRNAs across 16 tissue types and normal/cancer conditions. Here, we analyze vtRNA genes chromatin of the TCGA patient cohort withdrawn from two approaches: the assay for transposase-accessible chromatin followed by NGS sequencing (ATAC-seq) to analyze chromatin accessibility ( Buenrostro *et al.*, 2013; Buenrostro *et al.*, 2015; Corces *et al.*, 2018; Thurman *et al.*, 2012) and the Illumina Infinium Human Methylation 450K BeadChip to analyze the CpG methylation of DNA ( Moran *et al.*, 2016). This led us to determine the patterns of transcriptional regulation of the four human vtRNAs throughout cancer tissues. We also evaluate the association between vtRNA promoter DNA methylation and the patient clinical outcome in all the available cancer types. Finally, looking for functional discoveries, we analyze vtRNA relations with immune subtypes and transcriptionally co-regulated gene programs. Our study reveals specific patterns of inferred expression for each vtRNA that support previous evidence and poses potential new roles and molecular programs in which they may participate across and intra cancer types, increasing the comprehension of the role of the human vtRNAs in health and disease.

## Methods

### ATAC-seq data

The ATAC-seq data obtained by The Cancer Genome Atlas (TCGA) consortium were retrieved from UCSC Xena Browser ( Goldman *et al.*, 2020) on 03/10/2018. It comprises the genomic matrix TCGA_ATAC_peak_Log2Counts_dedup_promoter with normalized count values for 404 samples (385 primary solid tumor samples across 23 tissues). As is described in UCSC Xena Browser to calculate the average ATAC-seq values a prior count of 5 was added to the raw counts, then put into a "counts per million", then log2 transformed, then quantile normalized; the result is the average value in the file (log2(count+5)-qn values) across all technical replicates and all biospecimens belonging to the same TCGA sample group. The gene promoter for ATAC-seq is defined as a region within -1000 to +100bp from the TSS site. Assignment of promoter peak to gene mapping information derives from the peak summit within the promoter region. Peak location information of the ATAC-seq values for 500 bp gene promoter regions was retrieved from the file TCGA_ATAC_Log2Counts_Matrix.180608.txt.gz. All the analyses were performed for tissues with at least five primary tumor samples (21 tissues), which resulted in the exclusion of CESC (2 primary tumor samples) and SKCM (4 primary tumor samples). Correlations between ATAC-seq and DNA promoter methylation were performed for the 329 primary tumors that are studied by both strategies ( *Extended Data*: Tables S1 and S2).

### DNA methylation data

The DNA methylation data obtained from The Cancer Genome Atlas (TCGA) consortium was retrieved from UCSC Xena Browser ( Goldman *et al.*, 2020) on date 03/10/2018. It comprises the normalized beta-value of DNA methylation obtained using Illumina Infinium Human Methylation 450 BeadChip arrays of the total (9149 samples) normal adjacent tissues (746 samples across 23 tissues) and primary solid tumors (8403 samples across 32 tissues) of the Pan-Cancer TCGA cohort. The current study is limited to solid tumors since only AML DNA methylation is available at the TCGA. All the analyses were performed for tissues with at least five samples. Normal adjacent tissues comprise 16 tissues, excluding CESC (two samples), GBM (two samples), PCPG (three samples), SARC (four samples), SKCM (two samples), STAD (two samples) and THYM (two samples). Primary tumors comprise 32 tissues. The normalized promoter average beta-values (500 bp bin) comprise the following CpG sites for vtRNA1-1: cg12532653, cg05913451, cg14633504, cg16615348, cg25602765, cg13323902, cg18296956; for vtRNA1-2: cg21161173, cg13303313, cg00500100, cg05174942, cg25984996, cg11807153, cg15697852; for vtRNA1-3: cg23910413, cg19065177, cg02053188, cg01063759, cg07379832, cg07741016; and for vtRNA2-1: cg16615357, cg08745965, cg00124993, cg26896946, cg25340688, cg26328633, cg06536614, cg18678645, cg04481923 ( *Extended Data*: Tables S2–S5).

### Analysis of transcription factors associated to vtRNA genes

The transcription factors (TFs) occupancy analysis of vtRNAs was performed at a ± 3000 bp region centered at each vtRNA gene. The CHIP-seq data was retrieved from the UCSC genome browser (GRCh37/hg19) ( Kent *et al.*, 2002) and the K562 chronic myelogenous leukemia cell line was chosen since it has the richest TFs data of ENCODE dataset (ENCODE Transcription Factor Binding tracks - ENCODE 3 TFBS) ( Euskirchen *et al.*, 2007). The KEGG pathways enrichment analysis of the core 23 TFs common to all vtRNAs was done using STRING software ( Szklarczyk *et al.*, 2017) ( *Extended Data*: Table S6).

### Overall survival analysis

The Kaplan Meier curve analysis ( Bland & Altman, 1998) was performed with software GraphPad Prism 6 using Log-ranked Mantel-Cox test. We analyzed the ATAC-seq normalized values and DNA methylation average normalized beta-values for vtRNA promoters and survival data until 4000 days (99% of data). The log-rank statistics was calculated for quartiles of vtRNAs promoter chromatin accessibility comparison groups (25 ^th^ and 75 ^th^) ( *Extended Data*: Tables S7 and S8).

### Pathway enrichment and cluster chromosome localization analyses

The genome wide correlation analysis of promoter chromatin accessibility (ATAC-seq normalized values) for each vtRNA in comparison with all the annotated genes was performed using the genomic matrix TCGA_ATAC_peak_Log2Counts_dedup_promoter retrieved from UCSC Xena Browser (385 primary tumor samples across 23 tissues) ( Goldman *et al.*, 2020) on date 03/10/2018. The protein coding genes showing a Spearman correlation r ≥ 0.4 were selected for pathway and cluster location analysis. The pathway enrichment analysis was done using STRING software for the Gene Ontology Biological Process and KEGG pathways categories ( Szklarczyk *et al.*, 2017). The cluster localization analysis was performed with two approaches, the Cluster Locator algorithm ( Pazos Obregón *et al.*, 2018) and the Enrichr software ( Kuleshov *et al.*, 2016) ( *Extended Data*: Tables S9 and S10).

### Immune subtypes data

The data of Immune Subtypes defined by Thorsson *et al.* ( Thorsson *et al.*, 2018) is available at the Pan-Cancer TCGA and was retrieved from UCSC Xena Browser ( Goldman *et al.*, 2020) on date 03/10/2018 and from the supplementary material of Thorsson *et al.* article ( Thorsson *et al.*, 2018). ATAC-seq data of vtRNA promoters for the six Immune Subtype comprised a total of 364 samples: wound healing (105 samples), IFN-g dominant (93 samples), inflammatory (110 samples), lymphocyte depleted (43 samples), immunologically quiet (nine samples), TGF-b dominant (four samples). DNA methylation average normalized beta-values of vtRNA promoters for the six Immune Subtype comprised 7693 samples including wound healing (1963 samples), IFN-g dominant (2137 samples), inflammatory (2126 samples), lymphocyte depleted (933 samples), immunologically quiet (383 samples), TGF-b dominant (151 samples) ( *Extended Data*: Tables S11–S14).

### Statistical analysis

Unless specified all the variables are expressed as average value ± standard deviation (SD). Statistical analyses were performed with one-way ANOVA for multiple comparison tests, including Sidak’s Honest Significant Difference test as a post-hoc test, as referred in the legend of the Figures. Spearman equations were applied to determine the correlations. All the analyses were done in R software (version 3.6) using libraries ggcorrplot, corrplot, xlsx, heatmap.2 (clustering distance measured by Euclidean and Ward clustering algorithms) and software GraphPad Prism 6. The statistical significance of the observed differences was described using the p-value (* p < 0.05, ** p < 0.01, *** p < 0.001, **** p-value < 0.0001). Differences with a p-value of < 0.05 were considered significant.

## Results

### VtRNA genes have different chromatin accessibility at the promoter region

The Cancer Genome Atlas consortium incorporated ATAC-seq data for tumor samples of the GDC Pan-Cancer dataset (385 primary tumors samples across 23 cancer types ( Corces *et al.*, 2018)). The ATAC-seq strategy is a genome-wide sequencing profiling approximation to chromatin accessibility using the hyperactive Tn5 transposase that simultaneously cut and ligate adapters preferentially to the accessible DNA regions. The counts of sequencing reads in a particular genomic region provides a direct measurement of chromatin accessibility ( Buenrostro *et al.*, 2015; Tsompana & Buck, 2014). For those gene promoters regulated by DNA methylation, ATAC-seq is a more direct approximation to chromatin accessibility than promoter CpG DNA methylation measurements because the latter is well correlated but not an exact measurement of chromatin accessibility. Indeed, the effect of DNA methylation on chromatin structure depends on the gene and the distribution of the methylation sites along it. It is also important to mention that gene expression interpretations based solely on chromatin accessibility are blind to post-transcriptional regulatory events such as processing, localization, and stability of the RNA transcript. Nevertheless, since the chromatin status of the different vtRNA promoters has been positively correlated with the abundance of their transcripts ( Ahn *et al.*, 2018; Fort *et al.*, 2018; Helbo *et al.*, 2015; Lee *et al.*, 2014a; Lee *et al.*, 2014b; Park *et al.*, 2017a; Park *et al.*, 2017b; Sallustio *et al.*, 2016; Treppendahl *et al.*, 2012), ATAC-seq is likely a good surrogate of vtRNA expression. DNA CpG methylation data is available for 746 normal adjacent (across 23 tissues) and 8403 primary tumor (across 32 tissues) tissue samples of the Pan-Cancer TCGA cohort ( *Extended Data*: Tables S1–S5 and Figure S1). The full data is listed in Tables S1–S5 and the tissue composition of the three datasets of the cohort, comprising tumor ATAC-seq, normal and tumor DNA methylation, is shown in Figure S1. The DNA methylation data has nine more tissue types and a higher number of samples than the others, while the abundance of some tissues is skewed to twice enrichment in 1-4 specific tissue types.

Although several reports demonstrated that the DNA methylation of vtRNA promoters is well correlated to vtRNA transcript expression in various tissues ( Ahn *et al.*, 2018; Fort *et al.*, 2018; Helbo *et al.*, 2015; Helbo *et al.*, 2017; Lee *et al.*, 2014a; Lee *et al.*, 2014b; Park *et al.*, 2017b; Sallustio *et al.*, 2016; Treppendahl *et al.*, 2012), the association between the DNA methylation and chromatin accessibility at their promoters was not previously investigated.

**Figure 1.**
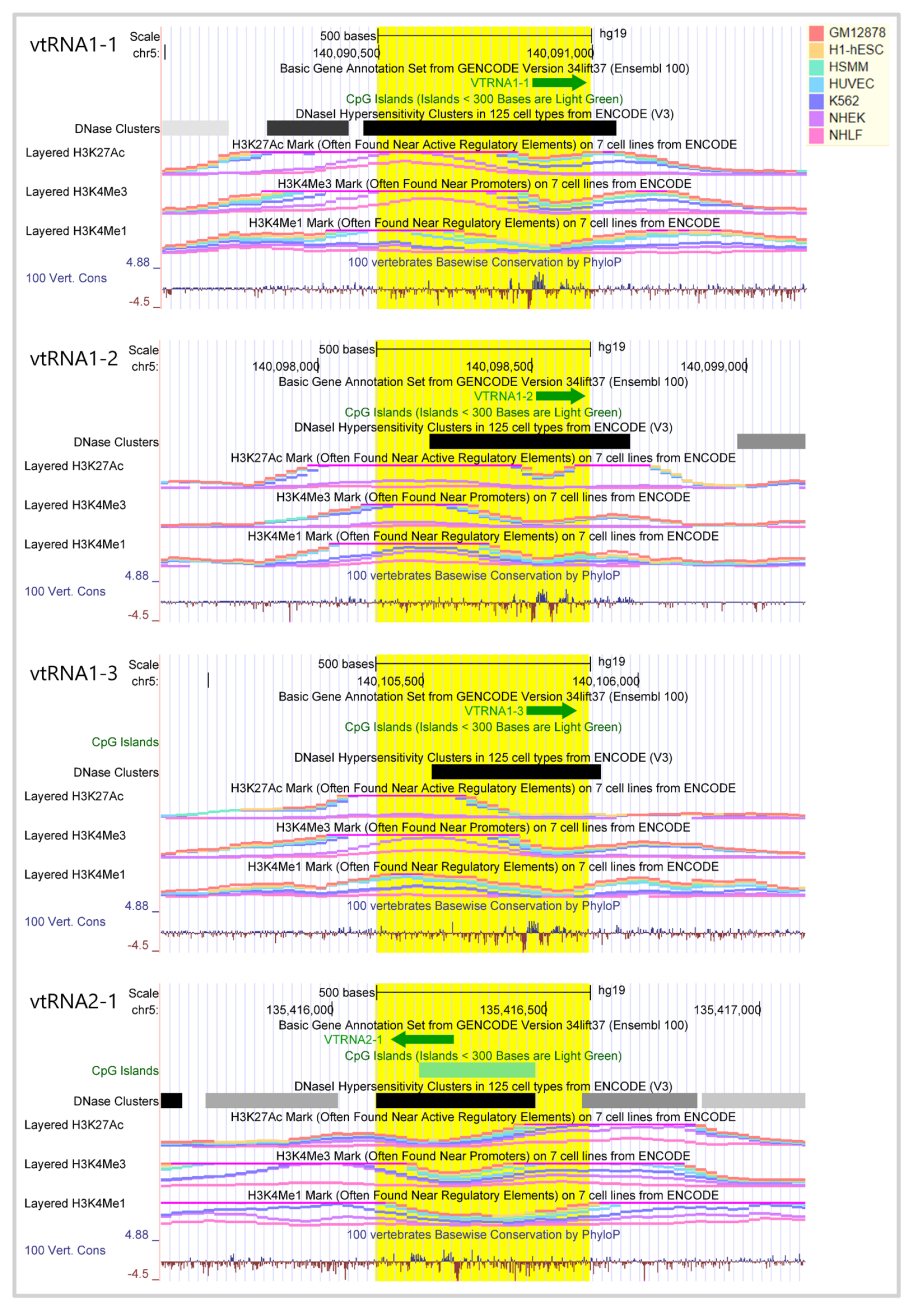
Genomic view of human vtRNA genes epigenetic features. Genomic view of 1.5 kb region of human vtRNA genes in UCSC Genome browser (GRCh37/hg19) centered at the 500bp bin highlighted in *yellow,* which was used for ATAC-seq and CpG methylation analyses. VtRNAs of locus 1 cluster (vtRNA1-1, vtRNA1-2, vtRNA1-3) and of locus 2 (vtRNA2-1) are in the sense and antisense orientation respectively. Several Gene annotation and ENCODE Project tracks for seven cell lines (GM12878, H1-hESC, HSMM, HUVEC, K562, NHEK, NHL) are displayed: DNA accessibility (DNaseI hypersensitivity clusters (color intensity is proportional to the maximum signal strength)), DNA methylation (for CpG islands length greater than 200 bp), histone modification (H3K27Ac, H3K4me1, H3K4me3 marks), conservation of the region in 100 Vertebrates (log-odds Phylop scores). The vertical viewing range of the tracks displays the default settings of the browser for each variable.

**Figure 2.**
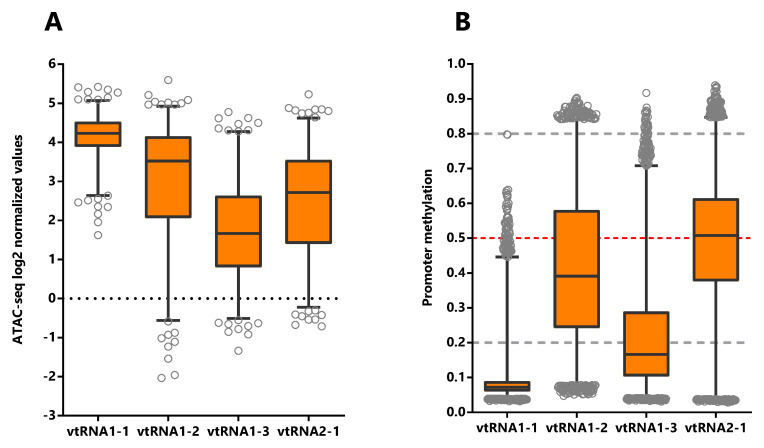
Chromatin accessibility and DNA methylation of the vtRNA promoters. Analysis of vtRNA 500bp promoter region. **A.** Average chromatin accessibility ATAC-seq values for the 385 primary tumors available at the Pan-Cancer TCGA dataset (23 tissues types). **B.** Average DNA methylation beta-values for the 8403 primary tumors (32 tissues types). Dashed horizontal lines denote unmethylated (bottom gray, average beta-value ≤ 0.2), 50% methylated (middle red, average beta-value = 0.5) and highly methylated (top gray, average beta-value ≥ 0.8) promoters. The box plots show the median and the lower and upper quartile, and the whiskers the 2.5 and 97.5 percentile of the distribution.

To assess the expression of vtRNAs in cancer tissues, we analyzed a 500 bp region, containing the full vtRNA genes, the proximal promoter region and the RNA polymerase lII A and B box elements located downstream of the transcription start site (TSS) ( Figure 1). Indeed, Vilalta *et al.*, 1994 demonstrated that the deletion of the 300bp 5´-flanking region of the rat vtRNA transcript largely reduced its transcription greater than 30-fold ( Vilalta *et al.*, 1994). As depicted in Figure 1, this region bears epigenetic marks of transcriptionally active chromatin ( Li *et al.*, 2007), including histone H3K27ac, H3K4me1, H3K4me3 at the promoters and DNaseI hypersensitivity clusters around the TSS in the cell types compiled by UCSC browser ( Goldman *et al.*, 2013). Interestingly, vtRNA2-1 is the only vtRNA immersed in a CpG island and vtRNA1-1 displays the strongest epigenetic marks of transcriptional activity ( Figure 1). The analysis of the ATAC-seq values of this 500 bp region of all the TCGA primary tumors reveals that the four human vtRNAs have different levels of chromatin accessibility ( Figure 2A). Moreover, the average DNA methylation of the promoters ( Figure 2B) in primary tumor samples supports this finding (32 tissues, *Extended Data*: Figure S1 and Tables S2 and S4). VtRNA1-1 promoter has the highest chromatin accessibility and the lowest DNA methylation, showing the smallest dispersion of values among the samples (Ave. 4.1, 0.6 SD) relative to the other three vtRNAs. Meanwhile, vtRNA1-2 (Ave. 2.9, 1.6 SD), vtRNA1-3 (Ave. 1.7, 1.2 SD) and vtRNA2-1 (Ave. 2.5, 1.4 SD) have broader ranges of ATAC-seq and DNA methylation values ( Figure 2, Table 1, *Extended Data*: Tables S1 and S2 and Figure S1). A comparison among the vtRNAs shows that the average chromatin accessibility of their promoters is inversely proportional to their average DNA methylation ( Table 1 and Figure 2) (relative ATAC-seq values are vtRNA1-1 (4.1) > vtRNA1-2 (2.9) > vtRNA2-1 (2.5) and accordingly, relative DNA methylation are vtRNA1-1 (0.09) < vtRNA1-2 (0.4) < vtRNA2-1 (0.5)). Yet vtRNA1-3 average low chromatin accessibility is not accompanied by a comparatively denser promoter methylation (values 1.7 and 0.22 respectively). Nevertheless, the intra-sample correlation between the ATAC-seq and DNA methylation for the vtRNAs is between -0.24 and -0.78 (see rs-values in Table 1 and *Extended Data*: Figure S2), suggesting that regardless of the relatively smaller average effect of DNA methylation on vtRNA1-3 promoter accessibility, all the vtRNAs are regulated by DNA methylation to some extent ( Table 1). In addition, the CpG DNA methylation negatively correlates with ATAC-seq values in the 500bp of the promoters of the four vtRNAs ( Table 1 and *Extended Data*: Figure S2). The strength of the correlation for each vtRNA is higher for the two vtRNAs that have a broader spectrum of DNA methylation, i.e. vtRNA1-2 and vtRNA2-1 (rs = -0.74 and rs = -0.78 respectively) ( Table 1 and *Extended Data*: Figure S2B and S2D). Meanwhile, vtRNA1-1 and vtRNA1-3 promoters, which have narrower range of DNA methylation (vtRNA1-1 has also a small range of chromatin accessibility) present a smaller association between both values (rs = -0.24 and rs = -0.54 respectively) ( Table 1 and *Extended Data*: Figure S2A and S2C). Technical differences in ATAC-seq and DNA methylation array approaches, as well as naturally occurring non-linear correlation between average DNA methylation and chromatin structure may account for these differences ( Corces *et al.*, 2018; Liu *et al.*, 2018). The low ATAC-seq value of the pseudogenes vtRNA3-1P (Ave. -1.2, 0.6 SD) and vtRNA2-2P (Ave. 0.1, 0.6 SD), represents a proof of concept of the analyses (Extended Data: Figure S3 and Tables S1-S5). The same approach was performed for the Y-RNAs genes RNY1 (Ave. 3.7, 0.4 SD), RNY3 (Ave. 2.1, 0.6 SD, RNY4 (Ave. 1.9, 0.6 SD) and RNY5 (Ave. 3.1, 0.4 SD) ( Canella *et al.*, 2010), which code for ubiquitously expressed RNA polymerase III transcripts ( *Extended Data*: Figure S3 and Tables S1).

**Table 1.** Correlation between vtRNA promoters ATAC-seq and DNA methylation values in the Pan-Cancer TCGA samples.

	Average ± SD		Spearman correlation (ATAC-seq vs DNA methylation)	
vtRNA	Promoter ATAC-seq	Promoter DNA methylation	*Rs*	*p-value*
**vtRNA1-1**	4.1 ± 0.6	0.09 ± 0.07	-0.24	<0.0001
**vtRNA1-2**	2.9 ± 1.6	0.42 ± 0.20	-0.74	<0.0001
**vtRNA1-3**	1.7 ± 1.2	0.22 ± 0.15	-0.54	<0.0001
**vtRNA2-1**	2.5 ± 1.4	0.47 ± 0.21	-0.78	<0.0001

The Spearman correlation was calculated for the 329 primary tumors studied by ATAC-seq and DNA methylation.

### Chromatin accessibility, DNA methylation and transcription factor binding at the four vtRNA promoters are correlated

**Figure 3.**
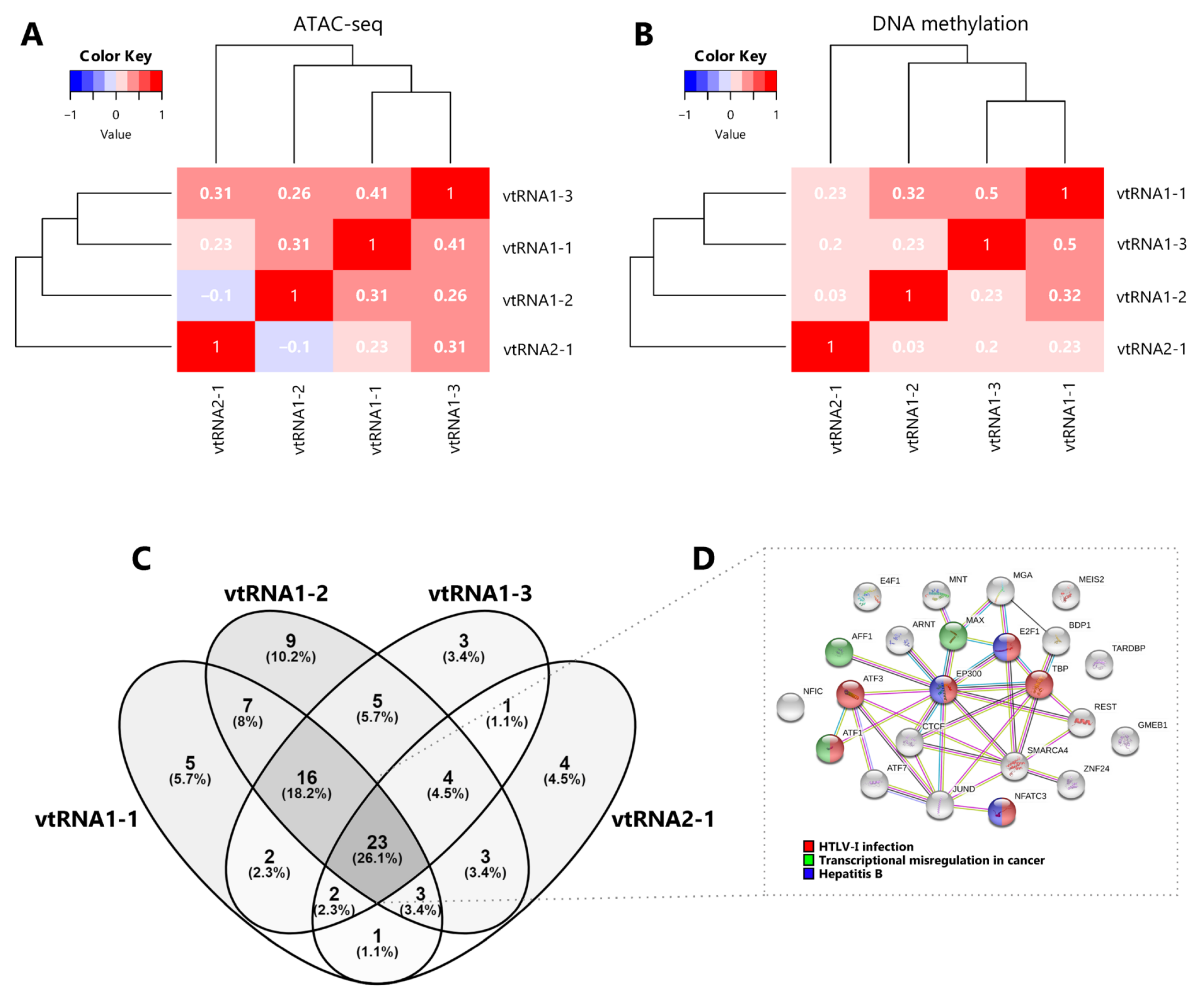
Comparative chromatin accessibility and transcription factor occupancy at the vtRNA loci. **A–B** Matrix of pairwise Spearman correlations (two-way hierarchical clustering distance measured by Euclidean and Ward clustering algorithms) of vtRNA 500 bp promoter region for ATAC-seq data (385 primary tumors samples across 23 tissues) ( **A**) and DNA methylation average beta-values data (8403 primary tumors samples across 32 tissues) ( **B**). **C.** Venn diagram of transcription factors identified as ChIP-seq Peaks by ENCODE 3 project in the cell line K562 (Venny 2.1; https://bioinfogp.cnb.csic.es/tools/venny/index.html). The region for TFs assignment was defined as ±3000 bp from the vtRNA transcript sequence boundaries. **D.** Interaction cluster of the core 23 TFs common to all vtRNAs performed with STRING ( Szklarczyk *et al.*, 2017). The colored labels of the top 3 enriched KEGG pathway terms (FDR < 0.05) are indicated.

Aiming to investigate a possible transcriptional co-regulation of vtRNA genes, we determined the pairwise correlations in chromatin accessibility and DNA methylation among the four vtRNA promoters. We found that all pairs of vtRNAs, except vtRNA1-2 and vtRNA2-1, show positive correlations in both datasets ( Figure 3A and 3B) (rs-values 0.23-0.41 for ATAC-seq and rs-values 0.20-0.50 for DNA methylation). The unsupervised clustering of these epigenetic marks indicates that the three vtRNAs clustered at vtRNA1 locus (vtRNA1-1, vtRNA1-2 and vtRNA1-3), which are the vault particle associated vtRNAs, are more similar than vtRNA2-1 ( Figure 3A and 3B).

Given that the chromatin accessibility of a DNA region is partially controlled by transcription and chromatin remodeler’s factors ( Corces *et al.*, 2018; Klemm *et al.*, 2019), we analyzed transcription factors (TFs) occupancy at a ± 3000 bp region centered at each vtRNA gene. Since there is no such study of the TCGA samples, we investigated the CHIP-seq data of the K562 chronic myelogenous leukemia cell line, which has the richest TF data at ENCODE dataset ( Euskirchen *et al.*, 2007; Goldman *et al.*, 2013). We observed that the four vtRNAs share a common core of 23 TFs (26%). The three members of the vtRNA1 cluster share 16 additional TFs (18%), reaching 39 common TFs (44%), while only 2-4 TFs with vtRNA2-1 ( Figure 3C and 3D and *Extended Data*: Table S6). We finally asked whether this common core of TFs is linked to a specific biological process. The STRING enrichment analysis of the core of 23 TFs common to all vtRNAs identified HTLV-I infection (ATF1, ATF3, E2F1, EP300, NFATC3, TBP), Hepatitis B (E2F1, EP300, NFATC3) and Transcriptional misregulation in cancer (AFF1, ATF1, MAX) as the top three enriched KEGG pathway terms (FDR < 0.05) ( Figure 3D and *Extended Data*: Table S6).

Taken together these findings indicate that the transcriptional activity of the vtRNAs is gene specific, being vtRNA1-1 the most accessible and possibly the most expressed. In addition, the data suggest that the regulatory status of the vtRNA promoters could be coordinately controlled, and the three vtRNA1s are more co-regulated among themselves. Yet, the core of TFs shared by the four vtRNAs is associated with viral infection and cancer related terms in the myeloid cell line K562.

### The vtRNA promoters present gene and tissue specific patterns of chromatin accessibility in primary tumors

**Figure 4.**
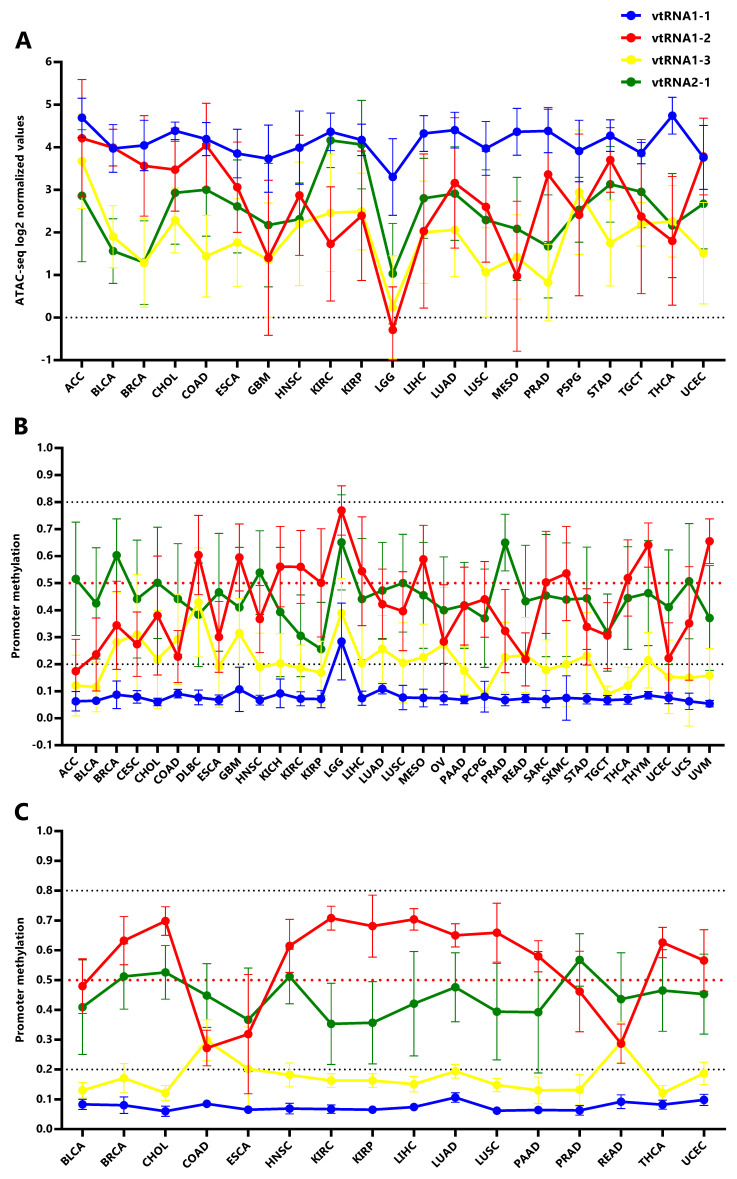
Chromatin accessibility and DNA methylation of the vtRNA promoters in tumor and normal tissues. **A.** ATAC-seq values for vtRNA promoters in 21 primary tumor tissues. **B.** Average beta-values of promoter DNA methylation for vtRNAs in 32 primary tumors tissues. **C.** Average beta-values of promoter DNA methylation for vtRNAs in 16 normal adjacent tissues. The charts show the average value and standard deviation of each vtRNAs for the tissues with at least five samples available at Pan-Cancer TCGA dataset.

In order to investigate the expression of vtRNAs in different tissues we analyzed the Pan-Cancer TCGA ATAC-seq data discriminating the tissue of origin. As expected, vtRNA1-1 has the highest promoter chromatin accessibility among tissues and the smallest variation ( Figure 4A, *Extended Data*: Tables S1-S5 and Figure S1). Remarkably, low-grade glioma tumor samples (LGG) show a global reduction in chromatin accessibility at the four vtRNA gene promoters ( Figure 4A). An opposite pattern is seen in adrenocortical carcinoma tissue (ACC), where the vtRNAs have the highest concerted chromatin accessibility ( Figure 4A). Individually, vtRNAs promoter accessibility is maximum and minimum in THCA (4.7) and LGG (3.3) for vtRNA1-1, ACC (4.2) and LGG (0.29) for vtRNA1-2, ACC (3.7) and LGG (0.24) for vtRNA1-3 and KIRC (4.2) and LGG (1.0) for vtRNA2-1 ( Figure 4A). VtRNA3-1P reaches a maximum of promoter chromatin accessibility in Testicular Germ Cell Tumors (TGCT) (0.65) with an extremely low average ATAC-seq value and its minimum in PRAD (-1.5) ( *Extended Data*: Figure S3C). VtRNA2-2P reaches a maximum and minimum promoter chromatin accessibility in Lung Adenocarcinoma (LUAD) (1.0) and ACC (-0.34) respectively ( *Extended Data*: Figure S3C). Remarkably, although vtRNA1-1 and vtRNA1-3 have the highest and lowest promoter accessibility in the majority of tissues respectively, vtRNA1-2 and vtRNA2-1 are the most variable (vtRNA1-1 SD = 0.34, vtRNA1-2 SD = 1.4, vtRNA1-3 SD = 0.75 and vtRNA2-1 SD = 0.78) ( Figure 4A). In addition, in 12 tissues the chromatin accessibility of vtRNA1-2 is higher than vtRNA2-1 (57%) whereas in nine tissues the chromatin accessibility of vtRNA1-2 is lower than vtRNA2-1 (43%) ( Figure 4A).

Using the vtRNAs promoter DNA methylation data, we extended the analysis to 32 primary tumor tissues, incorporating nine more tissues than ATAC-seq samples (DLBC, KICH, OV, PAAD, READ, SARC, THYM, UCS, UVM) ( Figure 4B, *Extended Data*: Figure S1) and 16 normal adjacent tissues ( Figure 4C, *Extended Data*: Figure S1). Again, the DNA methylation profile mirrors the chromatin accessibility in the individual tissue types ( Figure 4). Remarkably, the association between the average vtRNA promoter chromatin accessibility and DNA methylation of the vtRNA1 cluster in each tissue type is higher than that observed for the average of all tissues (vtRNA1-1 rs = -0.28, vtRNA1-2 rs = -0.90 vtRNA1-3 rs = -0.74 and vtRNA2-1 rs = -0.58) ( Table 1 and *Extended Data*: Figure S4). The opposite finding for vtRNA2-1 may be explained by the impact of the previously recognized epigenetic variation in the locus ( Silver *et al.*, 2015), which may prevail over the tissue specific variation for this gene.

In primary tumor tissues, promoter DNA methylation of vtRNA2-1 is higher than vtRNA1-2 in 17 tissues (53%) and the opposite is observed in the remaining 15 tissues (47%) ( Figure 4B). Meanwhile in normal tissues, promoter DNA methylation of vtRNA2-1 is higher than vtRNA1-2 in 4 tissues (25%) and lower in 12 tissues (75%) ( Figure 4C), indicating that although vtRNA1-2 is more methylated in normal samples, vtRNA2-1 gains methylation in neoplastic tissue, becoming more methylated than vtRNA1-2 (in 7 tissue types and vtRNA1-2 loose methylation: BLCA, BRCA, CHOL, HNCSC, LUAD, LUSC, UCEC). Indeed, the Fisher's exact test identifies a mirrored profile of chromatin accessibility between normal (4 tissues with high vtRNA1-2 and 12 tissues with high vtRNA2-1 from 16 total tissues) and tumor tissues (17 tissues with high vtRNA1-2 and 15 tissues with high vtRNA2-1 from 32 total tissues) for vtRNA1-2 and vtRNA2-1 (p-value = 0.07). Despite this deregulation in transformed tissues, we wondered if the tissue specific differences in vtRNA promoters were explained by their pre-existing status in the normal tissues. The correlation among average vtRNA promoter DNA methylation in normal and tumor tissues suggest that the variation in chromatin accessibility among tissue types is already established in the normal tissue counterparts (vtRNA1-1 rs = 0.53, vtRNA1-2 rs = 0.84, vtRNA1-3 rs = 0.47 and vtRNA2-1 rs = 0.75, *Extended Data*: Figure S5).

### VtRNA promoter´s DNA methylation is associated with tumor stage and tissue of origin

**Figure 5.**
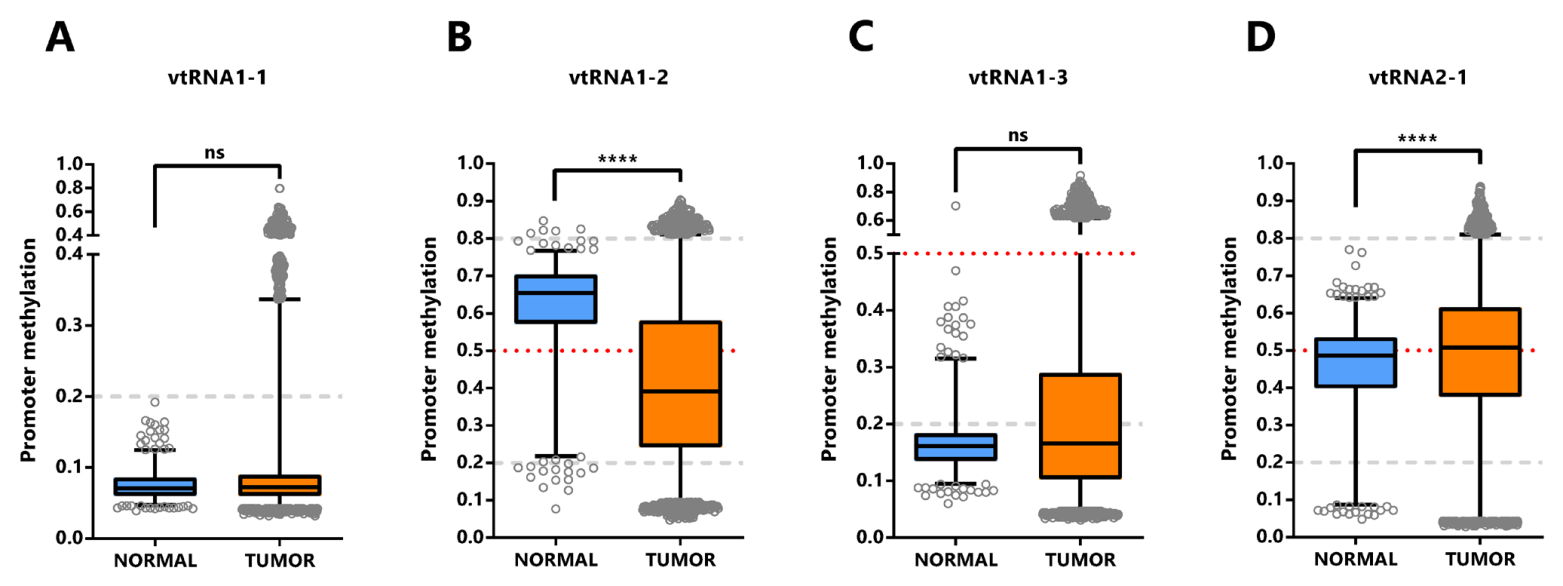
Promoter DNA methylation of vtRNAs in Normal and Tumor tissues of Pan-Cancer TCGA dataset. Average beta-values of promoter DNA methylation of vtRNA1-1 ( **A**), vtRNA1-2 ( **B**), vtRNA1-3 ( **C**) and vtRNA2-1 ( **D**), assessed in 746 normal and 8403 tumor tissues respectively. The box plots show the median line and lower and upper quartile and the whiskers the 2.5 and 97.5 percentile. Horizontal grey striped and red dotted lines denote unmethylated (average beta-value ≤ 0.2), 50% methylated (average beta-value = 0.5) and highly methylated (average beta-value ≥ 0.8) promoters. One-way ANOVA multiple test analysis with Sidak as posthoc test was performed. **** p-value < 0.0001.

The assessment of differential vtRNA expression from normal to tumor condition at the TCGA cohort can only be performed using DNA methylation dataset since no ATAC-seq analyses were performed in the normal tissues. The average beta-value of CpG sites in the 500bp vtRNAs promoter of normal and tumor tissue evidenced gene specific patterns of deregulation in neoplastic tissues ( Figure 5). As depicted in Figure 4, vtRNA1-1 and vtRNA1-3 promoter DNA methylation and chromatin accessibility is low and high tissue-wide respectively, in both normal and tumor tissues (yet, there are highly methylated tumor outliers as LGG, that lack normal counterpart) ( Figure 4 and Figures 5A and 5C). Conversely, vtRNA1-2 and vtRNA2-1 revealed significant differences in average promoter DNA methylation among the tissue categories ( Figures 5B and 5D). In agreement with previous results, the methylation of vtRNA1-2 promoter decreases from normal to tumor samples, while vtRNA2-1´s increases ( Figures 5B and 5D and Figure 4).

**Figure 6.**
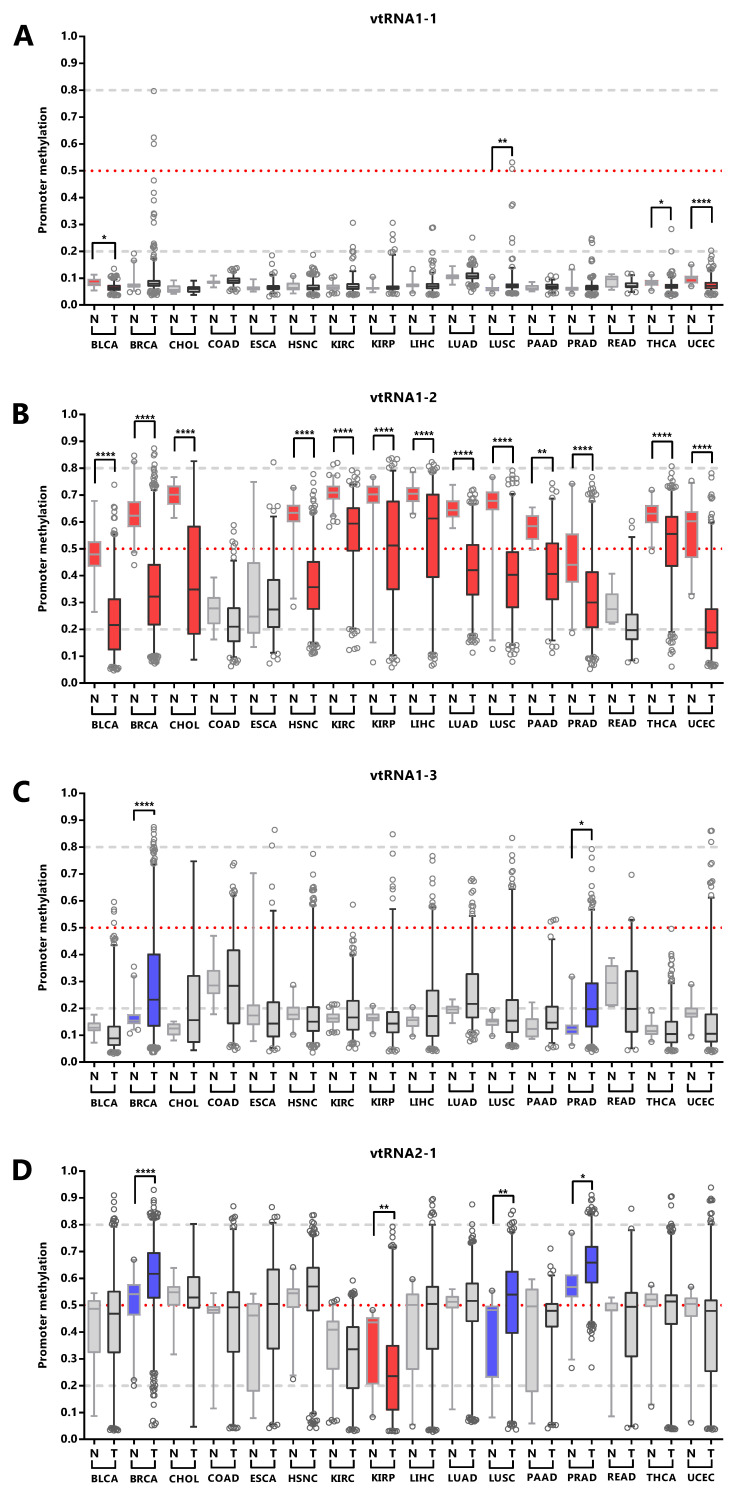
VtRNA promoters DNA methylation in Normal vs. Tumor samples. Average beta-values of promoter DNA methylation for vtRNA1-1 ( **A**) vtRNA1-2 ( **B**), vtRNA1-3 ( **C**) and vtRNA2-1 ( **D**). Acronyms indicate the tissue condition (normal (N) and tumor (T)). *Blue* and *red colors* indicate an increase or reduction in promoter methylation in tumor vs their normal tissues counterparts. The box plots show the median and the lower and upper quartile, and the whiskers the 2.5 and 97.5 percentile of the distribution. Horizontal, lines denote the methylation level of the promoters: grey striped bottom and top for unmethylated (average beta-value ≤ 0.2) or highly methylated (average beta-value ≥ 0.8) respectively, and red dotted for 50% methylated (average beta-value = 0.5)) promoters. One-way ANOVA multiple test analysis with Sidak as posthoc test was performed. * p-value < 0.05; ** p-value < 0.01; **** p-value < 0.0001.

We next asked whether the promoter status of the vtRNAs is deregulated during the normal and tumor transformation of different tissue types. This comparison was restricted to the 16 tissues with normal samples (with data of at least five samples, Figure 6 and *Extended Data*: Figure S1, Table S5). The results revealed that vtRNA1-1 is globally unmethylated in normal and tumor from different tissue origins, but presents small but statistically significant changes in 4 tissue types (OG profile in BLCA, THCA UCEC and TSG in LUSC), whose biological impact remains to be determined ( Figure 6A). Similarly, vtRNA1-3 is globally unmethylated, showing larger variability and significant upregulation in BRCA and PRAD, compatible with a TSG function (TSG trends for LIHC p-value = 0.08 and LUSC p-value = 0.09, Figure 6C). In agreement with the literature, vtRNA2-1 presents a TSG pattern of deregulation in PRAD, LUSC and BRCA ( Cao *et al.*, 2013; Fort *et al.*, 2018; Romanelli *et al.*, 2014), and an OG pattern in KIRP that has not been previously described ( Figure 6D). Finally, a statistically significant dysregulation of vtRNA1-2 promoter DNA methylation across almost all cancer types (13 out of 16 tissues analyzed) poses it as a candidate OG in cancer ( Figure 6B).

Overall, the data shows different DNA methylation changes in the vtRNAs promoters upon malignant transformation in several tissues. In addition, the deregulation of each vtRNA occurs mostly in a gene specific direction, where vtRNA1-2 has an OG like epigenetic de-repression while vtRNA2-1 (and to a lesser extent vtRNA1-1, vtRNA1-3) has a TSG like repressive methylation in tumor tissues.

### Low DNA methylation at the promoter of vtRNA1-1, vtRNA1-2 and vtRNA2-1 is associated with low patient overall survival

**Figure 7.**
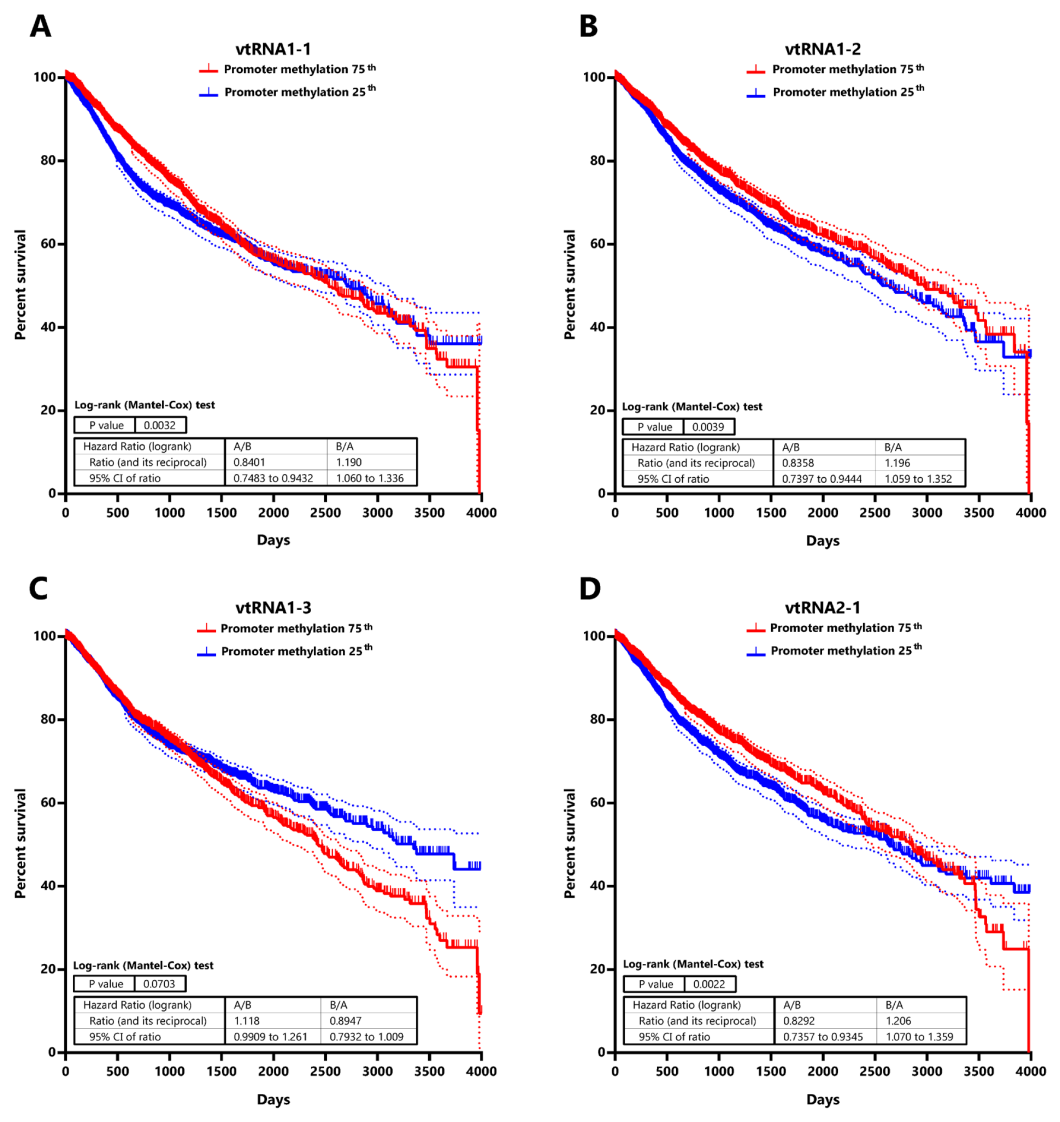
Association between patient Overall survival and vtRNA promoters DNA methylation status in Pan-Cancer TCGA dataset. Kaplan-Meier curves of overall patient survival probability over the time (4000 days) discriminating the primary tumors into two cohorts with relatively higher (75 ^th^) and lower (25 ^th^) DNA methylation at each vtRNA promoter. Promoter DNA methylation data of the 8060 primary tumors was used to stratify the patients in two quartiles, based on the expression of vtRNA1-1 ( **A**) vtRNA1-2 ( **B**), vtRNA1-3 ( **C**) and vtRNA2-1 ( **D**). Patient survival probability of the two groups was analyzed using Log-rank (Mantel-Cox) test. The dotted lines represent the 95% confidence interval for each curve.

Seeking for a possible clinical significance of the chromatin accessibility change of the vtRNA promoters, we studied its association with the overall survival of patients ( Figure 7 and *Extended Data*: Tables S7 and S8). The analysis of patients stratified by vtRNA promoter accessibility quartiles did not show differences in overall survival (data available at *Extended Data*: Table S8). However, a significantly lower patient survival probability when patient tumors have lower promoter DNA methylation is observed for vtRNA1-1 (p-value = 0.003), vtRNA1-2 (p-value = 0.004) and vtRNA2-1 (p-value = 0.002) (patient stratified in quartiles of the promoter DNA methylation cohort values, Figure 7). Therefore, a lower promoter DNA methylation of vtRNA1-1, vtRNA1-2 and vtRNA2-1, a surrogate of their high expression, might be associated with poor patient overall survival cancer-wide.

### Genome-wide chromatin accessibility correlations with vtRNA promoters reveal their link to specific cancer related functions

Seeking to get insight into the function of the vtRNAs from their transcription regulatory marks, we searched for the genes co-regulated at chromatin level. We calculated the correlation of the ATAC-seq promoter values of each vtRNA and all the individual genes in the 385 primary tumor cohort and selected those showing a Spearman correlation rs ≥ 0.4. Using STRING software analysis, we then investigated their connection with Biological Process categories and KEGG pathways ( Szklarczyk *et al.*, 2017). A few enriched terms for the genes correlated with vtRNA1-2, vtRNA1-3 and vtRNA2-1 is identified (FDR < 1×10 ^-3^) ( *Extended Data*: Tables S9 and S10). The 196 protein coding genes correlated with vtRNA1-2 are enriched in the Biological Process “Epithelial cell differentiation” (FDR < 1×10 ^-4^) ( *Extended Data*: Table S10). Meanwhile, the 358 protein coding genes correlated with vtRNA2-1 are enriched in the Biological Processes “Immune system process”, “Inflammatory response” and “Immune response” (FDR < 1×10 ^-5^) and the KEGG pathways term “Cytokine-cytokine receptor interaction” (FDR < 1×10 ^-4^) ( *Extended Data*: Table S10). The only six protein coding genes correlated with vtRNA1-3 are enriched in the “Thyroid cancer” KEGG pathway term (FDR < 1×10 ^-4^). Remarkably, although STRING analysis of the vtRNA1-1 associated 37 protein coding genes did not find enriched process or pathways, 36 of the 37 correlated genes are located at chromosome 5q region. Using the Cluster Locator algorithm ( Pazos Obregón *et al.*, 2018), we found a statistically significant non-random clustering behavior for the chromosomal location of the genes co-regulated with vtRNA1-1 (p-value < 1×10 ^-10^). Indeed, when the analysis was extended to 173 protein coding genes with Spearman correlation rs ≥ 0.3, 139 genes of 173 were situated at chromosome 5q region (p-value < 1×10 ^-10^). These genes are positioned in particular regions at chr5q31, chr5q35, chr5q23, chr5q14, chr5q32, chr5q34 (FDR < 1×10 ^-4^) ( Kuleshov *et al.*, 2016) ( *Extended Data*: Table S10). We did not find a similar phenomenon for the other three vtRNAs. In summary, vtRNA1-3, vtRNA1-2 and vtRNA2-1 transcription may be co-regulated with genes belonging to specific biological processes located elsewhere, while vtRNA1-1 transcription may be controlled by a locally specialized chromatin domain at chromosome 5q region lacking apparent functional relatedness.

### Immune Subtypes associated with the vtRNAs profile

**Figure 8.**
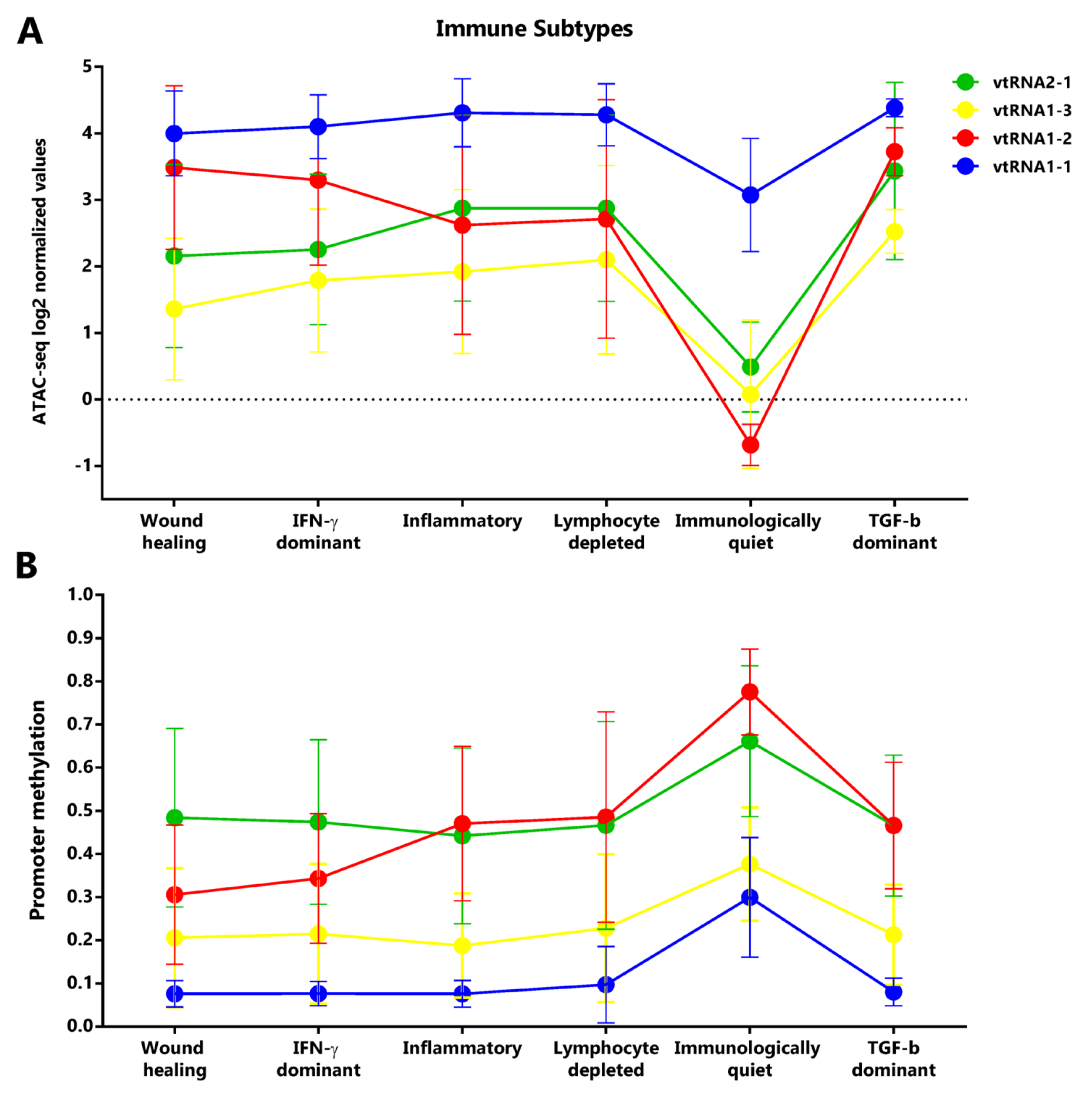
Chromatin accessibility and DNA methylation of vtRNA promoters in the Six Immune Subtypes analyzed in the PANCAN TCGA dataset. **A.** ATAC-seq values expressed as log2 normalized values for vtRNA promoters grouped in six Immune Subtype: wound healing (105 samples), IFN-g dominant (93 samples), inflammatory (110 samples), lymphocyte depleted (43 samples), immunologically quiet (9 samples), TGF-b dominant (4 samples). **B.** DNA methylation average beta-values for vtRNA promoters grouped in six Immune Subtype: wound healing (1963 samples), IFN-g dominant (2137 samples), inflammatory (2126 samples), lymphocyte depleted (933 samples), immunologically quiet (383 samples), TGF-b dominant (151 samples) ( Thorsson *et al.*, 2018). The charts show the average and standard deviation for each vtRNA promoter in each Immune Subtype group.

Taking into account that vtRNAs have been previously related to the immune response ( Golec *et al.*, 2019; Li *et al.*, 2015; Nandy *et al.*, 2009), and that we found an association of vtRNA2-1 with immune related terms, we sought to investigate a putative relation between the vtRNAs expression and the six Immune Subtypes defined by Thorsson *et al.* ( Thorsson *et al.*, 2018), compiled at Pan-Cancer TCGA ( *Extended Data*: Tables S11-S14). These six Immune Subtypes are named because of the foremost immune characteristic, comprising wound healing, IFN-g dominant, lymphocyte depleted, inflammatory, immunologically quiet and TGF-b dominant types ( Thorsson *et al.*, 2018). As was expected, mirrored patterns were observed for promoter ATAC-seq and DNA methylation data of the six groups ( Figure 8). The immunologically quiet subtype shows a concerted shutdown of all vtRNA promoters ( Figure 8), which is largely composed by LGG samples showing low vtRNA ATAC-seq and high promoter methylation average values in LGG tumors ( Figures 4A and 4B). The immunological quiet subtype exhibits the lowest leukocyte fraction (very low Th17 and low Th2) and the highest macrophage:lymphocyte ratio (high M2 macrophages) ( Thorsson *et al.*, 2018). The remaining subtypes present a similar vtRNA profile except for the inversion of vtRNA1-2/vtRNA2-1 accessibility ratio, which is higher than 1 for the wound healing and IFN-g dominant subtypes while is lower than 1 for the lymphocyte depleted and inflammatory subtypes ( Figure 8A). As expected, a mirrored pattern was observed for vtRNA1-2 and vtRNA2-1 at DNA methylation data ( Figure 8B). Since the wound healing and IFN-g dominant subtype have high proliferation rate opposite to the lymphocyte depleted and inflammatory subtypes ( Thorsson *et al.*, 2018), we evaluated the correlation between vtRNA1-2 and vtRNA2-1 chromatin status and proliferation rate or wound healing status defined by Thorsson *et al.* ( *Extended Data*: Tables S12 and S14) ( Thorsson *et al.*, 2018). A negative correlation between vtRNA1-2 promoter DNA methylation and the proliferation rate (rs = -0.44) and wound-healing features of the tumors is revealed (rs = -0.48) ( *Extended Data*: Tables S12 and S14). These findings suggest that vtRNA transcriptional regulation may be associated with tumor immunity.

## Discussion

Although important insight into the vtRNA function has been recently gained, the landscape of vtRNA expression in human tissues and cancer was unknown, perhaps for the absence of vtRNA data in transcriptomic studies. Here we used ATAC-seq and methylation arrays data from the Pan-Cancer TCGA consortium ( Weinstein *et al.*, 2013), as surrogate variables to uncover the expression of vtRNAs across multiple cancer types. A limitation of our study is its blindness to post-transcriptional controls of vtRNA expression, such as RNA processing, transport, localization and/or stability. Indeed, there is evidence of vtRNA transcript regulation via RNA methylation by NSUN2, or via its association to other proteins like DUSP11, DIS3L2, SSB, SRSF2 and DICER ( Burke *et al.*, 2016; Hussain *et al.*, 2013; Kickhoefer *et al.*, 2002; Łabno *et al.*, 2016; Persson *et al.*, 2009; Sajini *et al.*, 2019). Nonetheless, the chromatin status of the vtRNA promoters has been correlated with the expression of their transcripts in several reports ( Ahn *et al.*, 2018; Fort *et al.*, 2018; Helbo *et al.*, 2015; Helbo *et al.*, 2017; Lee *et al.*, 2014a; Lee *et al.*, 2014b; Sallustio *et al.*, 2016; Treppendahl *et al.*, 2012). These evidences unequivocally stand for the use of chromatin structure as a suitable proxy for vtRNA expression.

An integrated genomic view of the regulatory features of the vtRNA genes compiled in UCSC genome browser indicates that the four vtRNAs have chromatin signs of active transcription in different cell lines and defines a proximal promoter regulatory region of approximately 500 bp ( Nikitina *et al.*, 2011; Vilalta *et al.*, 1994). High resolution chromatin accessibility mapping studies by NOMe-Seq of the vtRNA promoters in cell lines validate this assumption ( Helbo *et al.*, 2017). Particularly, vtRNA2-1 is singular for being immersed in a CpG island, for lacking the TATA box, DSE or PSE elements characteristic of the others vtRNA promoters, and for bearing a cAMP-response (CRE) element ( Canella *et al.*, 2010; Helbo *et al.*, 2017; Nandy *et al.*, 2009). Since ATAC-seq approach is currently the best high throughput method to determine the chromatin accessibility of a DNA region directly ( Chen *et al.*, 2016; Sun *et al.*, 2019; Tsompana & Buck, 2014), we interrogated the ATAC-seq data of TCGA samples as a surrogate marker of vtRNA expression ( Corces *et al.*, 2018). Although DNA methylation is a less direct and less accurate measurement of chromatin accessibility compared to ATAC-seq, 25 times more TCGA samples have been analyzed by DNA Methylation Arrays, including matched normal tissues samples, thus this dataset is of great worth for the current study. Our analyses revealed that vtRNA1-1 has accessible chromatin and small variation in all the evaluated tissues, which is consistent with its identity as the ancestor vtRNA gene and its significance as the major RNA component of the ubiquitous vault particle ( Stadler *et al.*, 2009; van Zon *et al.*, 2001). In addition, vtRNA1-2, vtRNA1-3 and vtRNA2-1 have lower chromatin accessibility at their promoters and a larger variation among the samples. As expected, the average DNA methylation assessed by microarrays is negatively correlated with the chromatin accessibility assessed by ATAC-seq. Therefore, the landscape of vtRNA chromatin status was corroborated by both approaches. Yet, the low chromatin accessibility of vtRNA1-3 promoter is not reflected by a concomitant high DNA methylation, suggesting that additional regulatory features of this gene, acting in trans or cis, play a major influence in its chromatin structure. As was reported by Helbo *et al.* using NOMe-seq assay (Nucleosome Positioning), the chromatin at vtRNA1-3 may be poised for transcription, and still require TFs to achieve physiological levels of transcription ( Helbo *et al.*, 2017).

The relative abundance of the four vtRNAs determined in the current study has been previously observed in various cell lines, including H157, H1299, CRL2741, WI-38 and NTera-2 ( Lee *et al.*, 2011), HSC CD34+, HL60 ( Helbo *et al.*, 2015), T24, colorectal RKO, human diploid fibroblasts IMR90 ( Helbo *et al.*, 2017) and MCF7 ( Chen *et al.*, 2018). Yet, others found different comparative levels of the vtRNAs in MCF7, SW1573, GLC4, 8226, KB, SW620, MCF-7, HeLa, HEK-293 and L88/5 ( van Zon *et al.*, 2001), CBL, BL2 and BL41 ( Nandy *et al.*, 2009), EBV-negative Burkitt's lymphoma cell lines and A549 ( Li *et al.*, 2015) and in LNCaP, PC3, DU145, RWPE-1, HEK, HeLa and MCF-7 ( Stadler *et al.*, 2009) cell lines, suggesting that the expression of vtRNAs may differ in laboratory cell lines compared to tissues. Alternatively, the methods used to quantify the vtRNAs are not comparable among the studies.

Seeking to investigate regulatory connections between the vtRNAs as a valuable tool to study their function, we determine the pairwise correlations between the promoters of the vtRNAs. The positive correlations found are compatible with a coordinated transcriptional control of the vtRNAs, that is greater for the three members of the vtRNA1 cluster (specially vtRNA1-1 and vtRNA1-3). The exception is the pair vtRNA1-2 and vtRNA2-1, whose chromatin accessibility and promoter methylation are not linked in the cohort and can be indeed negatively associated.

As an alternative approach to investigate the vtRNA transcriptional co-regulation, we analyzed transcription and chromatin remodeling factors associated with their proximal promoter region. It is worth to mention that RNA polymerase II TFs clustered near TSS of RNA polymerase III transcripts could modulate occupancy and transcription rate of RNA polymerase III through RNA pol II activity ( Campbell & White, 2014; Gerber *et al.*, 2020; Raha *et al.*, 2010; Zhang *et al.*, 2019). Specifically, RNA polymerase II could assist chromatin opening, thus allowing accessibility of RNA polymerase III or its associated factors in the human genome ( Raha *et al.*, 2010). Interestingly, the 23 TFs common to all vtRNAs are related to viral infections and cancer. In agreement with their higher chromatin correlation, the vtRNA1 clustered genes have a larger core of common TFs (39) in comparison with vtRNA2-1 (23 TFs). The divergence of the transcriptional control of vtRNA2-1 was previously recognized given its unique regulatory elements and the complex developmental methylation it undergoes ( Canella *et al.*, 2010; Carpenter *et al.*, 2018; Helbo *et al.*, 2017; Nandy *et al.*, 2009). The upregulation of the four vtRNAs upon viral infection has been reported for several viruses, so it is possible that this core of TFs participate in the coordinated expression of vtRNA during viral infection and related responses ( Amort *et al.*, 2015; Li *et al.*, 2015; Nandy *et al.*, 2009). These TFs regulate cell cycle and translational arrest as well as the inhibition of host innate immune response ( Attar & Kurdistani, 2017; Ertosun *et al.*, 2016; Horsley & Pavlath, 2002; Jean *et al.*, 2000; Persengiev & Green, 2003; Rohini *et al.*, 2018). Since these processes are also hallmarks of cancer, the dysregulation of the normal function of vtRNAs in viral response may provide a selective advantage for cancer cell to overcome the many sources of stress faced during neoplastic evolution. If that holds true, the core vtRNA TFs may be co-opted in the malignant context to tune vtRNA expression favoring tumor progression. In agreement with that hypothesis, ATF1 and ATF3 were associated with CREB response and increased cell viability ( Persengiev & Green, 2003). Likewise, Golec *et al.* showed that altered levels of vtRNA2-1 modulate PKR activation and consequently altered the levels of CREB phosphorylation during T cell activation, which is a prerequisite for IFN-g activity ( Golec *et al.*, 2019). Furthermore, E2F1, a cell cycle regulator, was described as a direct transcriptional regulator of vtRNA2-1 in cervical cancer cells ( Li *et al.*, 2017). Moreover, it was shown that MYC binding to vtRNA2-1 promoter raises its expression and could be the explanation for the increased levels of vtRNA2-1 in some tumors ( Park *et al.*, 2017a). Similarly, TGFB1 provokes the demethylation of vtRNA2-1 promoter and consequently increases its expression in ovarian cancer ( Ahn *et al.*, 2018).

Alternatively, since RNA polymerase III transcripts are involved in virus replication and immune response ( Graczyk *et al.*, 2015), it is possible that the TF bound to vtRNA vicinity are part of a global and not a vtRNA specific viral response.

Due to its importance for cancer biology, vtRNA2-1 transcriptional regulation was recently more investigated ( Yeganeh & Hernandez, 2020). Various studies reported tissue specific roles of vtRNA2-1 in normal ( Golec *et al.*, 2019; Lee *et al.*, 2019; Miñones-Moyano *et al.*, 2013; Sallustio *et al.*, 2016; Suojalehto *et al.*, 2014) and cancer tissues ( Ahn *et al.*, 2018; Fort *et al.*, 2018; Hu *et al.*, 2017; Im *et al.*, 2020; Jeon *et al.*, 2012a; Kunkeaw *et al.*, 2012; Lee *et al.*, 2011; Lee, 2015; Lee *et al.*, 2016; Lee *et al.*, 2014a; Lee *et al.*, 2014b; Lei *et al.*, 2017; Li *et al.*, 2017; Ma *et al.*, 2020; Treppendahl *et al.*, 2012) and epigenetic alterations of the locus have been described in different malignancies ( Ahn *et al.*, 2018; Cao *et al.*, 2013; Fort *et al.*, 2018; Helbo *et al.*, 2015; Helbo *et al.*, 2017; Joo *et al.*, 2018; Lee *et al.*, 2014a; Lee *et al.*, 2014b; Park *et al.*, 2017a; Romanelli *et al.*, 2014; Treppendahl *et al.*, 2012). Meanwhile, except for Kirsten Grønbæk group´s contributions on the chromatin characterization of vtRNA1-3 and vtRNA1-2 promoters ( Helbo *et al.*, 2015; Helbo *et al.*, 2017), little is known about the transcriptional regulation and expression of the other vtRNAs. Likewise, to our knowledge, the global landscape of vtRNA expression across different cancer types has not been investigated. Our study shows that, except for vtRNA1-1, which has a high promoter chromatin accessibility and low DNA methylation in all the tissues, the other vtRNA promoters exhibit different levels of chromatin accessibility and DNA methylation among the tissue types. The relative status of the chromatin in different tissues is accurately mirrored by both approaches in the tissues examined by ATAC-seq and Illumina DNA methylation arrays. Interestingly, the same analysis performed in the normal tissue samples reveals that the tissue specific variation in DNA methylation of vtRNA promoters in the neoplastic tissues is already established in their normal tissue counterparts, indicating that the regulation of vtRNA expression is tuned during normal development and cell differentiation.

 While there is no apparent concerted modulation of the chromatin accessibility of the vtRNA promoters in most tissues, ACC and LGG tumors seem to be the exception. Concordantly, a global hypomethylation of malignant ACC tumors ( Legendre *et al.*, 2016; Rechache *et al.*, 2012), and an aberrantly methylation processes associated to altered DNMTs activity in LGG tumors were reported ( Nomura *et al.*, 2019). However, more research is necessary to understand the molecular basis of these observations.

The differential expression of the vtRNAs from normal to primary tumor tissues revealed different patterns depending on tissue origin. The average DNA methylation of the vtRNA1-1 and vtRNA1-3 promoters is not significantly deregulated, whereas vtRNA1-2 and vtRNA2-1 are decreased and increased respectively in tumor tissues. The latter finding favors a candidate OG and TSG function of vtRNA1-2 and vtRNA2-1 in cancer, respectively. Indeed, the epigenetic repression of vtRNA2-1 was previously reported by Romanelli *et al.* in BLCA, BRCA, COAD and LUSC analyzing the same data from TCGA ( Romanelli *et al.*, 2014). Furthermore, functional studies in LUSC, PRAD, ESCA and AML provided experimental support to that hypothesis ( Cao *et al.*, 2013; Fort *et al.*, 2018; Lee *et al.*, 2014a; Treppendahl *et al.*, 2012). Yet, vtRNA2-1 has been also proposed as an OG in ovarian, thyroid, endometrial, cervical and renal cancer ( Ahn *et al.*, 2018; Hu *et al.*, 2017; Lee *et al.*, 2016; Lei *et al.*, 2017; Li *et al.*, 2017; Yeganeh & Hernandez, 2020). In agreement with the functional data found by Lei *et al.* ( Lei *et al.*, 2017), we found an epigenetic de-repression of vtRNA2-1 promoter DNA methylation in renal carcinoma (KIRP). Likewise, a closer look at THCA samples, shows an average decrease in the DNA methylation of vtRNA2-1 promoter that supports the OG function described in this tissue ( Lee *et al.*, 2016). Unfortunately, normal ovarian, cervical and endometrial tissues are not available at the TCGA. Finally, vtRNA1-2 has an oncogenic pattern of inferred expression between normal and tumor tissues for 13 of 16 tissues analyzed, raising a possible oncogenic function for this RNA.

Our study found also an association of DNA methylation at the promoter with a shorter overall survival for vtRNA1-1, vtRNA1-2 and vtRNA2-1. This is surprising for vtRNA2-1, since it has both TSG and OG roles and TSG compatible expression profiles in three cancer types, inferred by DNA methylation of its promoter in tumor vs normal tissues and the average promoter DNA methylation is increased in tumor compared to normal tissue. A higher impact of vtRNA2-1 expression in patient survival in the oncogenic context may justify this discrepancy. On the contrary, the association between vtRNA1-2 low promoter DNA methylation and low patient survival is in agreement with its OG expression in cancer. The lack of survival association with the ATAC-seq values may be due to the small number of patients studied by this approach.

VtRNA expression in individual cancer types, inferred from the epigenetic status of their promoters, has been associated with patient survival in several studies of vtRNA2-1, one of vtRNA1-3 and none for vtRNA1-1 or vtRNA1-2. Low methylation or high expression of vtRNA2-1 promoter were associated with good prognosis or overall survival in lung ( Cao *et al.*, 2013), esophageal ( Lee *et al.*, 2014a), prostate ( Fort *et al.*, 2018), AML ( Treppendahl *et al.*, 2012), gastric ( Lee *et al.*, 2014b) and liver ( Yu *et al.*, 2020). Conversely, a worse prognosis or overall survival association of vtRNA2-1 was reported in thyroid ( Lee *et al.*, 2016) and ovarian ( Ahn *et al.*, 2018). From these reports, only prostate ( Fort *et al.*, 2018) and gastric ( Lee *et al.*, 2014b) studies used the TCGA data. Likewise, the methylation status of the vtRNA1-3 promoter associates with overall survival in the lower risk Myelodysplastic Syndrome patients ( Helbo *et al.*, 2015). Additionally, vtRNA1-1 and vtRNA1-2 correlate to chemotherapeutic resistance by direct interaction with drugs (doxorubicin, etoposide and mitoxantrone) and the modulation of vtRNA1-1 confirmed this finding in osteosarcoma cell lines ( Gopinath *et al.*, 2005; Gopinath *et al.*, 2010; Mashima *et al.*, 2008; van Zon *et al.*, 2001). Furthermore, increased levels of vtRNA1-1 were associated with increased proliferation and chemoresistance due to GAGE6 induction in MCF-7 cells ( Chen *et al.*, 2018). Besides, Norbert Polacek group showed that vtRNA1-1 expression confers apoptosis resistance in several human cell lines (BL2, BL41, HS578T, HEK293, A549 and HeLa) and revealed it capacity to repressed intrinsic and extrinsic apoptosis pathway ( Amort *et al.*, 2015; Bracher *et al.*, 2020). The later findings are in agreement with the lower patient survival associated with high levels of vtRNA1-1 expression cancer-wide observed in our analysis.

Seeking to get insights into the cancer related function of the vtRNAs we performed a genome wide search for genes co-regulated at the level of promoter chromatin accessibility. Remarkably, we identified immune and cytokine related terms for the genes co-regulated with vtRNA2-1. This association agrees with its proposed roles in innate immune modulation via PKR repression or OAS1 regulation and in cytokine production ( Calderon & Conn, 2018; Golec *et al.*, 2019; Lee *et al.*, 2020; Li *et al.*, 2015). Furthermore, vtRNA2-1 has been associated with autoimmune disorders ( Renauer *et al.*, 2015; Weeding & Sawalha, 2018) and tumor engraftment in prostate cancer ( Ma *et al.*, 2020). This enrichment in viral infection pathways, together with the viral infection involvement of the core TFs common to all vtRNAs, reinforces the hypothesis of the re-utilization of a regulatory RNA induced upon viral response in favor of cancer development at upstream and downstream regulatory steps. Additionally, we found a non-random clustering localization at chromosome arm 5q for genes co-regulated with vtRNA1-1. The chromosome arm 5q and regions chr5q31 and chr5q32-q33 were found frequently deleted in myelodysplastic syndrome (MDS) and acute myeloid leukemia (AML) ( Fuchs, 2012; Treppendahl *et al.*, 2012). Furthermore, the modulation of vtRNA2-1 and vtRNA1-3 was previously associated with human bone marrow CD34+ cells, AML and MDS ( Helbo *et al.*, 2015; Treppendahl *et al.*, 2012). Yet, our analysis points to vtRNA2-1 and vtRNA1-2, and to a lesser extent to vtRNA1-1 and vtRNA1-3, as the most relevant drivers across cancer types.

Support for the involvement of the vtRNAs in the immune responses in the context of cancer came from the association of their chromatin status with the immune subtype categories defined by Thorsson *et al.* ( Thorsson *et al.*, 2018). Our findings suggest a possible role of the vtRNAs in lymphocyte response in tumor samples, since the immunologically quiet subtype shows the lowest leukocyte fraction and vtRNAs promoter chromatin accessibility ( Thorsson *et al.*, 2018). Meanwhile, the vtRNA2-1 and vtRNA1-2 expression distinguishes two groups among the remaining four subtypes. The participation of vtRNA2-1 in the induction of the IFN-g and IL-2 expression in activated T cells through PKR modulation ( Golec *et al.*, 2019) goes in agreement with this hypothesis. Although the same study demonstrated that vtRNA1-1 expression was unchanged during T cell activation, it is remarkable that the 5q31-q33 region, comprising the vtRNA1 locus and chromatin co-regulated genes, encodes several cytokines that regulate the differentiation of Th1 and Th2 lymphocytes and has been linked with susceptibility to infections ( Jeronimo *et al.*, 2007; Lacy *et al.*, 2000; Naka *et al.*, 2009; Rodrigues *et al.*, 1999). Furthermore, vtRNA2-1 levels were recently associated with macrophages M1/M2 fates in prostate PC3 cell line mice xenografts via TGF-b ( Ma *et al.*, 2020), two pathways that define the six immune subtypes ( Thorsson *et al.*, 2018). Finally, we found that the proliferation rate and wound healing of the tumors are associated with the levels of vtRNA1-2 chromatin accessibility, which reinforces an OG role of vtRNA1-2 in cancer. Nonetheless, the participation of the vtRNAs in immune cells response inside the neoplastic niche needs to be further investigated.

## Conclusion

Taken together our analysis reveals the pattern of chromatin accessibility and DNA methylation at the four vtRNA promoters, analyzed by tissue of origin in the TCGA cohort. The agreement between both dataset and previous related literature endorses the use of these variables as surrogates of the vtRNA transcripts expression. VtRNA genes are enriched in viral related TFs. The comparison of TFs and chromatin status of the vtRNAs suggest that vtRNA2-1 is likely the most independently regulated vtRNA locus. The pattern of chromatin accessibility and DNA promoter methylation in normal vs tumor tissue and the association with cancer cell pathways and patient survival suggest that vtRNA2-1 and vtRNA1-2 are possibly the more relevant contributors to cancer. The results favor tissue specific TSG and OG roles for vtRNA2-1, a still not investigated oncogenic role of vtRNA1-2 and a limited cancer driver effect of vtRNA1-1/3 in specific tissue types. Lastly, we uncovered new evidence linking the vtRNAs with the immune response, cell proliferation and overall survival in cancer, which guarantees further investigation.

## Data availability

### Underlying data

All raw data used in this study can be downloaded from Xena Browser, a tool to explore functional genomic data sets ( Goldman *et al.*, 2020) ( https://xenabrowser.net/), and UCSC genome browser ( Kent *et al.*, 2002) ( https://genome.ucsc.edu/).

### Extended data

Zenodo: Pan-Cancer chromatin analysis of the human vtRNA genes - Supplementary Figures, http://doi.org/10.5281/zenodo.4784353 ( Fort & Duhagon, 2021a)

This project contains the following extended data:

- **Figure S1.Data type and tissue sample distribution.** Sample distribution per tissue type in the ATAC-seq, DNA methylation, ATAC-seq & DNA methylation and Normal adjacent tissue sample groups. **A.** Number of samples. **B.** Percentage of samples. The color reference, the number of samples of this tissue and the accounted percentage are presented to each tissue. Names references: ACC (Adrenocortical carcinoma), BLCA (Bladder Urothelial Carcinoma), BRCA (Breast invasive carcinoma), CESC (Cervical squamous cell carcinoma and endocervical adenocarcinoma), CHOL (Cholangiocarcinoma), COAD (Colon adenocarcinoma), DLBC (Lymphoid Neoplasm Diffuse Large B-cell Lymphoma), ESCA (Esophageal carcinoma), GBM (Glioblastoma multiforme), HNSC (Head and Neck squamous cell carcinoma), KICH (Kidney Chromophobe), KIRC (Kidney renal clear cell carcinoma), KIRP (Kidney renal papillary cell carcinoma), LGG (Brain Lower Grade Glioma), LIHC (Liver hepatocellular carcinoma), LUAD (Lung adenocarcinoma), LUSC (Lung squamous cell carcinoma), MESO (Mesothelioma), OV (Ovarian serous cystadenocarcinoma), PAAD (Pancreatic adenocarcinoma), PCPG (Pheochromocytoma and Paraganglioma), PRAD (Prostate adenocarcinoma), READ (Rectum adenocarcinoma), SARC (Sarcoma), SKCM (Skin Cutaneous Melanoma), STAD (Stomach adenocarcinoma), TGCT (Testicular Germ Cell Tumors), THCA (Thyroid carcinoma), THYM (Thymoma), UCEC (Uterine Corpus Endometrial Carcinoma), UCS (Uterine Carcinosarcoma) and UVM (Uveal Melanoma).
- **Figure S2.Correlation between ATAC-seq values and promoter methylation values of vtRNAs in Pan-Cancer TCGA dataset.A–D.** The correlation between ATAC-seq and DNA methylation average normalized beta values was calculated for vtRNAs (vtRNAs1-1 ( **A**), vtRNA1-2 ( **B**), vtRNA1-3 ( **C**) and vtRNA2-1 ( **D**)), in 329 primary tumors samples. The box plots show the median line and lower and upper quartile and the whiskers the 2.5 and 97.5 percentile. Horizontal grey striped and red dotted lines are shown to denote unmethylated (average beta-value ≤ 0.2), 50% methylated (average beta-value = 0.5) and highly methylated (average beta-value ≥ 0.8) levels. Spearman correlation value and the best-fit line (red line) with 95% confidence bands (black dot lines) are shown.
- **Figure S3.Genomic context and chromatin accessibility of human vtRNA3-1P, vtRNA2-2P and RNYs (RNY1, RNY3, RNY4 and RNY5).A.** Genomic view of the 1.5 kb region of human vtRNA3-1P, vtRNA2-2P and RNYs (RNY1, RNY3, RNY4 and RNY5) genes in UCSC Genome browser (GRCh37/hg19) centered at the 500bp of the ATAC-seq. Highlighted in yellow is the 500bp ATAC-seq region used in the posterior analyses. The following Gene annotation and ENCODE Project tracks for seven cell lines (GM12878, H1-hESC, HSMM, HUVEC, K562, NHEK, NHL) are displayed: DNA accessibility (DNaseI hypersensitivity clusters (color intensity is proportional to the maximum signal strength)), DNA methylation (CpG islands length greater than 200 bp), histone modification (H3K27Ac, H3K4me1, H3K4me3 marks), conservation of the region in 100 Vertebrates (log-odds Phylop scores). The vertical viewing range of the tracks displays the default settings of the browser for each variable. **B**. The distribution of ATAC-seq values for of human vtRNA3-1P, vtRNA2-2P and RNYs (RNY1, RNY3, RNY4 and RNY5) in Pan-Cancer TCGA dataset (385 tumors samples across 23 cancer types) expressed as log2 normalized values. The box plots show the median and the lower and upper quartile, and the whiskers the 2.5 and 97.5 percentile of the distribution. C. ATAC-seq values for of human vtRNA3-1P, vtRNA2-2P and RNYs (RNY1, RNY3, RNY4 and RNY5) in 21 different tumors with at least 5 tumor samples available in Pan-Cancer TCGA dataset (385 tumors samples) expressed as log2 normalized values. The chart shows the average and standard error for each vtRNAs in each tumor type.
- **Figure S4.VtRNA promoter’s chromatin accessibility (ATAC-seq and DNA methylation) average values for tumor tissuesfrom Pan-Cancer TCGA dataset.** The average vtRNA promoters ATAC-seq and DNA methylation values for each primary tumor tissue type (21 tissues). VtRNA1-1 ( **A**), vtRNA1-2 ( **B**), vtRNA1-3 ( **C**) and vtRNA2-1 ( **D**). The Spearman correlation between averages ATAC-seq and DNA methylation values was calculated for each vtRNA. The chart shows the average and standard deviation for each tissue with at least five samples available at Pan-Cancer TCGA.
- **Figure S5. VtRNA promoter’s DNA methylation for normal and tumor tissues from Pan-Cancer TCGA dataset.** The average promoter DNA methylation beta-values of normal adjacent (Normal) and primary tumor (Tumor) tissues (16 tissue types) for vtRNA1-1 ( **A**), vtRNA1-2 ( **B**), vtRNA1-3 ( **C**) and vtRNA2-1 ( **D**) is shown. The Spearman (r) correlation between normal and tumor values was calculated. The chart shows the average and standard deviation for each tissue with at least five samples available at Pan-Cancer TCGA.

Zenodo: Pan-Cancer chromatin analysis of the human vtRNA genes - Supplementary Tables, http://doi.org/10.5281/zenodo.4784473 ( Fort & Duhagon, 2021b)

This project contains the following extended data:

- **Tables S1–S5.ATAC-seq and DNA methylation data for vtRNA promoters in primary tumors and normal adjacent tissues.***CSV spreadsheets*: Table_S1_ATAC-seq_data_500bp: All ATAC-seq data of vtRNAs promoter (500bp) data for primary tumor samples; Table_S2_DNA_methylation_500bp: All DNA methylation data and ATAC-seq data of vtRNAs promoter (500bp) data for total primary tumor and normal adjacent samples; Table_S3_DNA_methylation_NORMAL: All DNA methylation data of vtRNAs promoter (500bp) data for normal adjacent samples; Table_S4_DNA_methylation_TUMOR: All DNA methylation data of vtRNAs promoter (500bp) data for primary tumor samples; Table_S5_Normal_&_Tumor_matched: All DNA methylation data of vtRNAs promoter (500bp) data for primary tumor and normal adjacent samples.
- **Table S6.VtRNAs Transcription Factor Binding and KEEG enriched terms.***CSV spreadsheets*: Table_S6_Binding_Factors: Transcription factors identified in the cell line K562 as ChIP-seq Peaks by ENCODE 3 project and KEEG_terms: enriched KEGG pathway terms (FDR < 0.05).
- **Tables S7–S8.DNA methylation, ATAC-seq data and associated survival data for primary tumors.***CSV spreadsheets*: Table_S7_DNA-methylation_Survival_data: All DNA methylation data of vtRNAs promoter (500bp) and survival data for primary tumor samples; Table_S8_ATAC-seq_Survival_data: ATAC-seq data of vtRNAs promoter (500bp) and survival data for primary tumor samples.
- **Tables S9–S10.Correlation of ATAC-seq values between vtRNA and all genome promoters in primary tumor samples.***CSV spreadsheets*: Table_S9_ATAC-seq_gene_promoter_spearman_correlation: Spearman correlation values of all promoter genes and vtRNAs in primary tumors samples; Table_S10_vtRNAs_pathway_enrichment_and_cluster_chromosome_localization_analysis: vtRNAs vtRNA1-1, vtRNA1-2, vtRNA1-3 and vtRNA2-1 pathway enrichment and cluster chromosome localization data.
- **Tables S11–S14.ATAC-seq and DNA methylation data for vtRNA promoters in primary tumors and the associated Immune Subtypes data.***CSV spreadsheets*: Table_S11_Immune_Subtypes_DNA_methylation_data: All DNA methylation data of vtRNAs promoter (500bp) and Immune Subtypes data for primary tumor samples; Table_S12_Spearman_corr_vtRNAs_Immune_Subtypes_DNA_methylation_data: Spearman correlation values of DNA methylation data of vtRNAs promoter (500bp) and Immune Subtypes data for primary tumor samples; Table_S13_Immune_Subtypes_ATAC-seq_data: All ATAC-seq data of vtRNAs promoter (500bp) and Immune Subtypes data for primary tumor samples; Table_S14_Spearman_corr_vtRNAs_Immune_Subtypes_ATAC-seq_data: Spearman correlation values of ATAC-seq data of vtRNAs promoter (500bp) and Immune Subtypes data for primary tumor samples.

Data are available under the terms of the Creative Commons Zero "No rights reserved" data waiver (CC0 1.0 Public domain dedication).
