# Supplementary material for: Pan-cancer chromatin analysis of the human vtRNA genes uncovers their association with cancer biology
Source: F1000Res. 2021 Mar 5;10:182. [Version 1] doi: 10.12688/f1000research.28510.1 (PMC8287541; doi:10.12688/f1000research.28510.1)
Supplement: Peer review history

# Response

**Published:** 2021-06-16

**Authors:** Maria Ana Duhagon

**Affiliations:** Universidad de la Republica, Uruguay

***Reviewer:** Daniele Hasler, Biochemistry Center Regensburg (BZR), Laboratory for RNA Biology, University of Regensburg, Regensburg, Germany.*

***Reviewers’ comment:** The study by Fort and Duhagon estimates the expression of a class of non-coding (nc)RNAs termed 'vault RNAs' (vtRNAs) across different cancer types. Since vtRNAs might be underrepresented applying conventional RNA sequencing techniques, the authors infer vtRNAs expression by analyzing chromatin characteristics at promoter regions of vtRNA genes. In more detail, Fort and Duhagon use genome-wide chromatin accessibility data (ATAC-seq) as well as DNA methylation data sets from The Cancer Genome Atlas (TCGA) consortium to provide a comprehensive comparison between the four annotated vtRNA genes. By this approach, the authors determine the correlation between chromatin accessibility and DNA methylation of vtRNA promoters and compare their chromatin characteristics across different cancer types. Furthermore, Fort and Duhagon use publically available CHIP-seq datasets originating from a leukemia cell line to determine transcription factors (TFs) binding to vtRNA genes and identify a set of 23 core TFs common to all vtRNAs which might govern chromatin accessibility at these genomic sites.*

*Further analyses of the TCGA DNA methylation data sets provide evidence for vtRNA-specific deregulation upon malignant transformation in specific cancer types, as well as lower patient survival probability associated with reduced vtRNA promoter methylation. Finally, the authors determine genes that are co-regulated with vtRNAs in terms of chromatin accessibility and the also profile chromatin accessibility and DNA methylation of vtRNA promoters in six immune subtypes of the pan-cancer TCGA datasets.*

*VtRNAs are a class of ncRNAs with rather enigmatic functions, albeit they have been recently implicated in important cellular processes ^1^ ^,^ ^2^ and in cancer biology[ref-]. The work by Fort and Duhagon is the first pan-cancer study focusing on vtRNAs and it provides a nice and comprehensive overview for the potential function of the single vtRNA transcripts as tumor suppressors or oncogenes in specific cancer types.*

*The manuscript is well written, although some aspects could have been presented a bit more concisely. In particular, the authors dedicate special emphasis to the relative comparison between vtRNA genes (e.g. Figures 3A and 3B and result section related to Figures 4 and 8). The question arises whether a key message of the study is that specific vtRNA genes are up- or down-regulated in certain cancer conditions or whether a key point is that the relative expression levels of the four vtRNA genes are altered. If so, the authors should propose a model in the discussion section.*

**Authors response:** We are grateful for the evaluation of our manuscript entitled “Pan-Cancer chromatin analysis of the human vtRNA genes uncovers their association with cancer biology”, since we find these constructive comments rather pertinent and valuable. We tried to answer all the points raised by the Reviewer and hope it led us to the improved of the manuscript.

 It is true that the article is long. We believe the length is justified by the type of information it provides. In particular, the description of the patterns of vtRNA inferred expression involves four genes in 20 cancer types assessed by two methods, plus normal tissues data for DNA methylation.

 We think that the message of the study is fairly written in the abstract and conclusion, neither of which prioritize the inferred relative expression of the vtRNAs per se, but what in the inferred expression pattern is suggestive of the involvement of the four genes in cancer. The article aimed to use the chromatin status of the genes to withdraw their candidate roles in cancer tissue. In our view the figures mentioned by the Reviewer are about their pattern of expression in cancer and immune subtypes, regulation, and the derived functional implications of those findings. Nevertheless, the narrative requires a description of their relative abundance and change, while their comparison helps to visualize how different they are expressed and the implication for their function in normal and tumor tissue.

**Additional major points:**

***Reviewers’ comment:** The major limitation of this study is that vtRNA expression was not analyzed directly but was inferred from chromatin accessibility data (ATAC-seq) as well as DNA methylation data. Nevertheless, the authors honestly point out this limitation and provide convincing evidences from literature that support the validity of their study. Taken together, this approach sounds reasonable.*

*Nevertheless, the findings could be further strengthened by analyzing TCGA miRNA-seq datasets for the expression of vtRNA-derived small RNA fragments. This strategy has been applied in previous studies to infer the expression of snoRNAs ^4^ and tRNAs ^5^ across different cancer types, assuming that small processing products correlate with expression levels of the full-length transcripts. Indeed snoRNAs and in particular tRNAs are ncRNA with similar sequencing biases as vtRNAs.*

**Authors response:** We agree the small RNA sequencing analysis proposed by the Reviewer would be valuable to provide further support for the expression of the vtRNAs inferred from the chromatin status. Indeed, we have done it previously while studying snc886-3p, a microRNA-like RNA derived from vtRNA2-1 (published in Fort *et al*., 2020). There we showed that nc886 and snc886-3p expression (determined by qRT-PCR) negatively correlate with DNA methylation in six PrCa cell lines analyzed (r Spearman -0.71).

 Nevertheless, since vtRNAs derived fragments are not quantified with the TCGA small RNA workflow, we need to request access to protected controlled-access data - primary sequencing data (BAM or FASTQ files) to identify them. We understand that this approach and the subsequent data analyses exceed the extent of the current manuscript.

 Attempting to follow the Reviewer’s recommendation using readily available data, we analyzed the NCI-60 cell lines panel, whose genome DNA methylation and small RNA-seq data is published. Indeed, the 4 vtRNA are known to be subject to posttranscriptional processing at some extent (Hussain *et al*., 2013; Sajini *et al*., 2019). We found that vtRNA2-1 produces more specific small RNAs than the other three vtRNAs, thus arguing in favor of a specialization of vtRNA2-1 as a Dicer substrate, which may explain the better correlation between the precursor and the small RNAs derived from it (see Panel A of the figure attached to this response; *Figure_reviewer_response-I*. Zenodo. http://doi.org/10.5281/zenodo.4784690). In addition, vtRNA2-1 generates more than 30 times small RNAs that the other vtRNAs (see Panel B of the figure attached to this response). As anticipated, the expression of the small RNA fragments derived from vtRNA2-1 shows a better correlation with promoter methylation in the NCI-60 cell lines (r= -0.77). Meanwhile, the small RNAs derived from vtRNAs of cluster 1 are less abundant and less associated to promoter methylation (r= -0.19- -0.24) (see Panel C of the figure attached to this response).

 In addition, the average abundance of the small RNAs derived from the vtRNAs of cluster 1 correlates with the relative methylation of their promoters (small RNAs derived from vtRNA1-1> vtRNA1-2> vtRNA1-3 and promoter methylation vtRNA1-1< vtRNA1-2< vtRNA1-3) (see Panel B of the figure attached to this response), in agreement with the TCGA data presented in our manuscript. Since small RNAs derived from vtRNA2-1 are more abundant than those derived from the other three vtRNAs, the relative expression of its fragments does not correlate with the relative DNA methylation of the others.

 In summary, the analysis of NCI-60 cell lines, a panel comprising all tissue types, confirms that the abundance of the small RNAs derived from vtRNAs negatively correlates with their promoter methylation but indicated it may be useful just for vtRNA2-1. The latter, together with the low abundance of the small RNAs derived from the vtRNAs and their processing (Hussain *et al.*, 2013; Sajini *et al.*, 2019), suggest that the abundance of small RNAs in the TCGA small RNA-seq data may not be a good proxy for vtRNA expression.

 As discussed above, the post-transcriptional regulation of vtRNA seems to represent an additional layer of complexity of vtRNA biology that includes the processing into small RNAs. We speculate that only some of the fragments derived from specific vtRNAs may have functional significance, but it is likely reached by mechanisms different from those in which their precursors vtRNAs participate. We believe small RNAs derived from vtRNA are an interesting subject of investigation, requiring an independent study, which incorporates the biological mechanism in which they play a part.

***Reviewers’ comment:** Finally, in order to be more accurate about the limitation of the study mentioned by the Reviewer, we substituted the term “expression” for “chromatin status” or related terms when was appropriate.*

*The analysis of TF occupancy at vtRNA loci is, in my opinion, rather speculative and vague. Since vtRNAs are transcribed by RNA polymerase III (pol III), is there any clear and direct evidence from literature that the identified TFs regulate Pol III activity (except for the TFIIIA, TFIIIB and TFIIIC complexes)? Could the binding of these TFs rather regulate transcription of adjacent RNA polymerase II genes and not of vtRNAs? The authors might at least briefly touch these aspects in the discussion.*

**Authors response:** We welcome the Reviewers comment since we had a similar concern about the significance of these observations. Yet, we reasoned that the identification of viral related transcription factors (TFs) near vtRNA genes was a provocative finding in the context of their involvement in viral infection and innate immune response. In addition, RNA Pol III (Pol III) has been linked to the innate immune response at different levels, thus it is tempting to speculate that the Pol III regulated genes are coordinately regulated by viral related TFs dependently or independently of RNA Pol II (Pol II) action. 

 The identified TFs that are shared by the vtRNA (23 TFs) comprise TFs known to be part of the Pol III basal machinery (3/23), or to be direct (6/23) or indirect interactors (3/23) of Pol III machinery. This observation endorses their participation in the vtRNA genes transcription.

 There are no Pol II genes near or overlapping the vtRNA genes, thus this in an unlikely explanation for TF occupancy in the vicinity of the genes. Indeed, several lines of evidence have shown that Pol II and some of its specific TFs affect Pol III activity (White, 2011). The localization of the Pol II and its specific TFs (FOS, JUN, ETS1) near Pol III promoters (200bp upstream of TSS) in human cell lines, the global regulation by Pol II specific TFs (p53, MAF1, MYC, RB), the regulation of Pol III by Pol II activity, the sharing chromatin marks and the basal TFIIIB component TBP. The direct interaction between specific TFs and TFIIIB is also known to modulate Pol III transcription.

 It has been proposed that Pol II may assist RNA pol III activity by either binding, transcription, or stalling, contributing to make the chromatin more permissive for transcription by Pol III. Curiously, Raha et al demonstrated a reduction of the three vtRNAs of locus 1 after α-amanitin treatment and the co-localization of Pol III and Pol II near these genes (Raha *et al.*, 2010). Additional evidence of the Pol II regulation of specific Pol III transcribed genes has been published, including tRNAs (mediated by MAF1 (Gerber *et al.*, 2020), GAF1 (Rodríguez-López *et al.*, 2020), U6 RNA (OCT1, SP1, STAF, reviewed in (Hirsch *et al*., 2000)) Alu elements (mediated by YY1, SP1, MEG2, FOS, EP300, AP-1, (Zhang *et al.*, 2019 and references therein). Finally, the crosstalk between the two polymerases seems to be tissue and gene specific, suggesting that Pol II-Pol III crosstalk may be finer that previously envisioned.

 To amend the lack of discussion of this aspect we included the following sentence in the Discussion of the manuscript: *“It is worth to mention that RNA polymerase II TFs clustered near TSS of RNA polymerase III transcripts could modulate occupancy and transcription rate of RNA polymerase III through RNA pol II activity (Campbell and White, 2014; Gerber et al., 2020; Raha et al., 2010; Zhang et al., 2019). Specifically, RNA polymerase II could assist chromatin opening, thus allowing accessibility of RNA polymerase III or its associated factors in the human genome (Raha et al., 2010).”*

***Reviewers’ comment:** It would be also worth to determine whether deregulation (e.g. of any of the 9 vtRNA1-2-specific TFs or any of the 4 vtRNA2-1-specific TFs) correlates with the changes of vtRNA promoter DNA methylation in normal vs tumor samples (Figure 6). This would be more informative than the current conclusions concerning shared TFs between vtRNA loci and their co-regulated expression. Indeed, this analysis might help to understand, e.g., how the expression of a single vtRNA locus is regulated within the vtRNA1 cluster.*

**Authors response:** Following the Reviewer´s suggestion, we determined the correlation between vtRNAs chromatin accessibility and the specific TFs associated with vtRNAs (Table attached to this revision, *Table_TFs_Binding_Factors.csv* Zenodo. http://doi.org/10.5281/zenodo.4784690), but we did not find significant associations. It is worth to recall that TF activity is strongly regulated at the post-transcription level, thus TF abundance may not be a good indicator of TF activity. It might be also reasoned that TFs may be poised in an inactive state, to turn active upon the proper signalling, which might well be a viral invasion or an immune system response mediator. Weather this TF DNA occupancy is relevant in cancer remains to be dilucidated.

 While reviewing the literature to respond to this point it came to our attention that there is a link between RNApol III and innate immune response (Graczyk *et al.*, 2015), that could be an alternative explanation of the viral TF enrichment at the vicinity of vtRNAs. Therefore, we decided to introduce this phrase at the end of the discussion of this aspect: *“Alternatively, since RNA polymerase III transcripts are involved in virus replication and immune response (Graczyk et al., 2015), it is possible that the TF bound to vtRNA vicinity are part of a global and not a vtRNA specific viral response.”*

 In view of the above, in an attempt to reduce the conjectures derived from the TFs analysis we modified the abstract and the conclusion of the paper as explained below.

 Abstract: We substituted the sentence “Although the vtRNAs are co-regulated by transcription factors related to viral infection, vtRNA2-1 is the most independently regulated homologue.” for the following: “ *VtRNAs promoters are enriched in transcription factors related to viral infection. VtRNA2-1 is likely the most independently regulated homologue.”*

 Conclusion: We substituted the sentence “The four vtRNAs seem to be co-regulated at the transcriptional level, and TFs involved in viral infection are likely to take part in their coordinated transcription.” for the following: “ *VtRNA genes are enriched in viral related TFs. The comparison of TFs and chromatin status of the vtRNAs suggest that vtRNA2-1 is likely the most independently regulated vtRNA locus.”*

***Reviewers’ comment:** Interestingly, the chromatin profile of vtRNAs in brain lower grade glioma (LGG) indicates that vtRNA expression is strongly reduced in this condition. Does this effect hold true also for other classes of pol III transcripts (e.g. Y RNAs)? If so, this finding might point towards a general reduction of pol III activity in LGG.*

**Authors response:** In order to answer the Reviewer, we determined the ATAC-seq chromatin accessibility of RNYs (RNY1, RNY3, RNY4 and RNY5). As seen in the new Supplementary Figure 3 and modified Supplementary Table 1, the RNYs showed a different chromatin accessibility profile within different tissues, among them and compared to vtRNAs. Interestingly, RNY3 and RNY4 show a conspicuously higher chromatin accessibility in LGG compared with other tissues, which is opposite to the lower accessibility observed for the vtRNAs These observations raise a possible RNA pol III misregulation in LGG tumors, which must be gene specific to justify opposite outcomes for RNAY and vtRNAs. The modified Supplementary Figure 3 and modified Supplementary Table 1 are now included in the manuscript.

 To incorporate these findings in the text, the following sentence was included the results section: *“The same approach was performed for the Y-RNAs genes RNY1 (Ave. 3.7, 0.4 SD), RNY3 (Ave. 2.1, 0.6 SD, RNY4 (Ave. 1.9, 0.6 SD) and RNY5 (Ave. 3.1, 0.4 SD) (Canella et al., 2010), which code for produce ubiquitously expressed RNA polymerase III transcripts (Extended Data: Figure S3 and Tables S1).”.*

**Minor points:**

***Reviewers’ comment:*** *The authors should reformulate their statement in the introduction “VtRNA2-1 has also been shown to act as a microRNA precursor”, as the vtRNA2-1-derived fragment was not convincingly proven to be a bona fide microRNA and the hsa-miR-886 entry has been retracted in the current miRBase release 22.1 ^6^ .*

**Authors response:** Following the advice, we modified the sentences related to small RNAs derived from vtRNA2-1. “ *VtRNA2-1 has also been shown to generate small RNAs that could act like microRNAs in different tissues,…*”.

***Reviewers’ comment:*** *Please correct the typo on page 3 replacing “TGFB1” by “TGFBI”; i.e. Transforming Growth Factor Beta Induced (TGFBI) and not Transforming Growth Factor Beta 1 (TGF1).*

**Authors response:** We are sorry for the mistake; we have corrected the name of the gene.

***Reviewers’ comment:*** *Since lower patient survival of cancer patients was associated with lower vtRNA promoter methylation and not with higher promoter accessibility, the paragraph “High chromatin accessibility at the promoter of vtRNA1-1, vtRNA1-2 and vtRNA2-1 is associated with low patient overall survival” should be renamed. In light of the statement “DNA methylation is a less direct and less accurate measurement of chromatin accessibility compared to ATAC-seq” (page 16), it is questionable whether the relationship between DNA methylation and patient survival is due to increased vtRNA expression, as a similar result would be expected also from the analysis of the ATAC-seq data. Is this simply due to a larger samples size of the DNA methylation dataset?*

**Authors response:** We amended the title of the paragraph to *“Low DNA methylation at the promoter of vtRNA1-1, vtRNA1-2 and vtRNA2-1 is associated with low patient overall survival.”*

 Despite no statistically significant differences in overall survival were observed using ATAC-seq patient data the curves were like those observed using the promoter DNA methylation. The lack of significance is likely due to the smaller ATAC-seq sample size as anticipated by the Reviewer. In addition, the association between OS and vtRNA promoter DNA methylation is weak per se, so sample size is likely critical to sense significant differences.

***Reviewers’ comment:*** *The authors do not mention that a pseudogene of vtRNA2-1 (vtRNA2-2P) has been annotated at chr2:65,555,432-65,555,534. Is there any reason for that? VtRNA2-2P could be included as proof of concept similarly to vtRNA3-1P on page 8.*

**Authors response:** We have previously looked for vtRNA2-2P in ATAC-seq data but there is no ATAC-seq region determined for this gene in the TSS, so we decided to do not include it. However, we now extended the analysis to the ATAC-seq region nearest to the TSS of vtRNA2-2P. As can be seen in modified Supplementary Figure 3 and modified Supplementary Table 1, the vtRNA2-2P has a very low and stable pattern of ATAC-seq chromatin accessibility across the different tissues that agrees with its pseudogene annotation. These results were now included in the manuscript and the text was modified accordingly. In the introduction section we added: *“The vtRNA3-1P and vtRNA2-2P are two additional vault RNA genes situated in chromosome X and 2 respectively, but were classified as a pseudogenes due to the absence of expression in cell lines and the presence of silencing mutations at the promoter (Büscher et al., 2020; van Zon et al., 2001).”. *In the results section we added: *“The low ATAC-seq value of the pseudogenes vtRNA3-1P (Ave. -1.2, 0.6 SD) and vtRNA2-2P (Ave. 0.1, 0.6 SD), represents a proof of concept of the analyses (Extended Data: Figure S3 and Tables S1-S5). The same approach was performed for the Y-RNAs genes RNY1 (Ave. 3.7, 0.4 SD), RNY3 (Ave. 2.1, 0.6 SD, RNY4 (Ave. 1.9, 0.6 SD) and RNY5 (Ave. 3.1, 0.4 SD) (Canella et al., 2010), which code for produce ubiquitously expressed RNA polymerase III transcripts (Extended Data: Figure S3 and Tables S1).” *and *“VtRNA2-2P reaches a maximum and minimum promoter chromatin accessibility in Lung Adenocarcinoma (LUAD) (1.0) and ACC (-0.34) respectively (Extended Data: Figure S3C).”*

***Reviewers’ comment:*** *Page 3: “…the presence of silencing mutations at the B box element of its promoter B-box”. Since the organization of RNA polymerase III promoters has not been explained before, it would be better to keep this statement more general without mentioning the B-box.*

**Authors response:** To attend the Reviewer´s suggestion, we modified the sentence to *“the presence of silencing mutations at the promoter”.*

***Reviewers’ comment:*** *The abbreviation “PANCAN” in the title of the legend to Figure 8 was not used before in the text.*

**Authors response:** Due the Reviewer´s comment, we modified “PANCAN” for *“Pan-Cancer”.*

***Reviewers’ comment:*** *I am not sure whether the term “chromatin polymorphism” on page 10 is correct. The referenced publication refers to epigenetic variations.*

**Authors response:** Due the Reviewer´s comment, we modified the term “chromatin polymorphism” for “ *epigenetic variation*”.

**References:**

 Fort, R.S., Garat, B., Sotelo-Silveira, J.R., & Duhagon, M.A. (2020). vtRNA2-1/nc886 Produces a Small RNA That Contributes to Its Tumor Suppression Action through the microRNA Pathway in Prostate Cancer. Non-Coding RNA *6*, 7.

 Gerber, A., Ito, K., Chu, C.S., & Roeder, R.G. (2020). Gene-Specific Control of tRNA Expression by RNA Polymerase II. Mol. Cell *78*, 765–778.

 Graczyk, D., White, R.J., & Ryan, K.M. (2015). Involvement of RNA Polymerase III in Immune Responses. Mol. Cell. Biol. *35*, 1848–1859.

 Hirsch, H.A., Gu, L., & Henry, R.W. (2000). The Retinoblastoma Tumor Suppressor Protein Targets Distinct General Transcription Factors To Regulate RNA Polymerase III Gene Expression. Mol. Cell. Biol. *20*, 9182–9191.

 Hussain, S., Sajini, A.A., Blanco, S., Dietmann, S., Lombard, P., Sugimoto, Y., Paramor, M., Gleeson, J.G., Odom, D.T., Ule, J., *et al*. (2013). NSun2-mediated cytosine-5 methylation of vault noncoding RNA determines its processing into regulatory small RNAs. Cell Rep. *4*, 255–261.

 Raha, D., Wang, Z., Moqtaderi, Z., Wu, L., Zhong, G., Gerstein, M., Struhl, K., & Snyder, M. (2010). Close association of RNA polymerase II and many transcription factors with Pol III genes. Proc. Natl. Acad. Sci. U. S. A. *107*, 3639–3644.

 Rodríguez-López, M., Gonzalez, S., Hillson, O., Tunnacliffe, E., Codlin, S., Tallada, V.A., Bähler, J., & Rallis, C. (2020). The GATA Transcription Factor Gaf1 Represses tRNAs, Inhibits Growth, and Extends Chronological Lifespan Downstream of Fission Yeast TORC1. Cell Rep. *30*, 3240-3249.e4.

 Sajini, A.A., Choudhury, N.R., Wagner, R.E., Bornelöv, S., Selmi, T., Spanos, C., Dietmann, S., Rappsilber, J., Michlewski, G., & Frye, M. (2019). Loss of 5-methylcytosine alters the biogenesis of vault-derived small RNAs to coordinate epidermal differentiation. Nat. Commun. *10*.

 White, R.J. (2011). Transcription by RNA polymerase III: More complex than we thought. Nat. Rev. Genet. *12*, 459–463.

 Zhang, X.O., Gingeras, T.R., & Weng, Z. (2019). Genome-wide analysis of polymerase III–transcribed Alu elements suggests cell-type–specific enhancer function. Genome Res. *29*, 1402–1414.
